# Hepatic pannexin‐1 mediates ST2^+^ regulatory T cells promoting resolution of inflammation in *lipopolysaccharide*‐induced endotoxemia

**DOI:** 10.1002/ctm2.849

**Published:** 2022-05-20

**Authors:** Pusen Wang, Baojie Shi, Chunguang Wang, Yuanyuan Wang, Weitao Que, Zhongyi Jiang, Xueni Liu, Qianwei Jiang, Hao Li, Zhihai Peng, Lin Zhong

**Affiliations:** ^1^ Department of General Surgery, Shanghai General Hospital Shanghai Jiao Tong University School of Medicine Shanghai China; ^2^ Department of Critical Care Medicine, Shanghai General Hospital Shanghai Jiao Tong University School of Medicine Shanghai China; ^3^ Unit of Pathogenic Fungal Infection & Host Immunity, CAS Key Laboratory of Molecular Virology and Immunology Institut Pasteur of Shanghai, Chinese Academy of Sciences Shanghai China

**Keywords:** ATP, endotoxemia, IL‐33, Panx1, Tregs

## Abstract

Sepsis remains the most lethal infectious disease and substantially impairs patient prognosis after liver transplantation (LT). Our previous study reported a role of the pannexin 1 (PANX1)–interleukin‐33 (IL‐33) axis in activating innate immunity to protect against methicillin‐resistant *Staphylococcus aureus* infection; however, the role of PANX1 in regulating adaptive immunity in sepsis and the underlying mechanism are unclear. In this study, we examined the role of the PANX1–IL‐33 axis in protecting against sepsis caused by a gram‐negative bacterial infection in an independent LT cohort. Next, in animal studies, we assessed the immunological state of Panx1^−/‐^ mice with lipopolysaccharide (LPS)‐induced endotoxemia and then focused on the cytokine storm and regulatory T cells (Tregs), which are crucial for the resolution of inflammation. To generate liver‐specific Panx1‐deficient mice and mimic clinical LT procedures, a mouse LT model was established. We demonstrated that hepatic PANX1 deficiency exacerbated LPS‐induced endotoxemia and dysregulated the immune response in the mouse LT model. In hepatocytes, we confirmed that PANX1 positively regulated IL‐33 synthesis after LPS administration. We showed that the adenosine triphosphate‐P2X7 pathway regulated the hepatic PANX1–IL‐33 axis during endotoxemia *in vitro* and *in vivo*. Recombinant IL‐33 treatment rescued LPS‐induced endotoxemia by increasing the numbers of liver‐infiltrating ST2^+^ Tregs and attenuating the cytokine storm in hepatic PANX1‐deficient mice. In conclusion, our findings revealed that the hepatic PANX1–IL‐33 axis protects against endotoxemia and liver injury by targeting ST2^+^ Tregs and promoting the early resolution of hyperinflammation.

## Graphical abstract

- Pannexin 1 (PANX1) deficiency exacerbated lipopolysaccharide (LPS)‐induced endotoxemia and dysregulated the immune response.
- Adenosine triphosphate‐P2X7 pathway regulated the hepatic PANX1–interleukin‐33 (IL‐33)axis during endotoxemia.
- IL‐33 rescued LPS‐induced endotoxemia by increasing the numbers of liver‐infiltrating ST2+ Tregs and attenuating the cytokine storm.

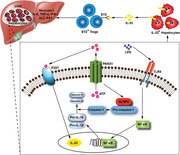

**Abbreviations**
AAV: adeno‐associated virus
ALT: alanine aminotransferase
AST: aspartate aminotransferase
ATP: adenosine triphosphate
DMEM: Dulbecco's modified Eagle medium
ELISA: enzyme‐linked immunosorbent assay
FBS: fetal bovine serum
GNB: gram‐negative bacteria
HE: hematoxylin and eosin
HRP: horseradish peroxidase
IL‐18: interleukin‐18
IL‐1β: interleukin‐1β
IL‐33: interleukin‐33
LPS: lipopolysaccharide
LT: liver transplantation
MRSA: methicillin‐resistant *Staphylococcus aureus*
NLRP3: NACHT, LRR and PYD domains‐containing protein 3
P2X: purinergic receptors type 2 X
PANX1: Pannexin‐1
PBS: phosphate‐buffered saline
rIL‐33: recombinant interleukin‐33
shRNA: short hairpin RNA
Tregs: regulatory T cells
TUNEL: terminal deoxynucleotidyl transferase‐mediated dUTP nick‐end labelling
WT: wild‐type

## 1 INTRODUCTION

Sepsis, which is one of the most lethal infectious diseases, substantially worsens the prognosis of patients treated with liver transplantation (LT).^1^, ^2^, ^3^, ^4^, ^5^ Studies have reported that LT recipients have an increased risk of sepsis compared to the general population of hospitalized patients.^5^, ^6^, ^7^ LT patients are susceptible to sepsis for many reasons, including cirrhosis‐associated immune dysfunction, changes in gut microbial composition, repeated hospital admissions, multiple antibiotic courses, bleeding and transfusion, invasive procedures, long intensive care unit stays, immunosuppressive therapies, and so on.^8^ According to the Sepsis‐3 criteria, up to 67% of patients develop sepsis after LT.^2^ Our previous study^9^ reported that the pannexin 1 (PANX1)‐IL‐33 axis is involved in methicillin‐resistant *Staphylococcus aureus* (MRSA) infection after LT, and this axis activates innate immunity, including recruiting macrophages and neutrophils, to protect against MRSA. However, it is unclear whether PANX1 also plays a role in regulating adaptive immunity in the defence against other infections, such as sepsis caused by gram‐negative bacteria (GNB) infection.

Traditionally, sepsis is thought to consist of an initial systemic, hyperinflammatory phase that occurs in response to infection followed by a hypoinflammatory phase that is characterized by anergy, lymphopenia, and secondary infections.^10^, ^11^ Currently, more studies have reported that the two phases occur simultaneously.^11^ The initial molecular response, also known as the cytokine storm, is the main cause of organ injury and death.^11^, ^12^ Studies have shown that an early resolution of inflammation and a return to homeostasis is beneficial in sepsis.^13^, ^14^ In LPS‐induced endotoxemia, resolution of the cytokine storm is crucial for the recovery of organ function; this is different from MRSA infection, in which the targeting and elimination of the pathogen are of great importance. In this study, we found that the PANX1–IL‐33 axis played a protective role in LPS‐induced endotoxemia by regulating host adaptive immunity and promoting the resolution of hyperinflammation.

PANX1 is a transmembrane glycoprotein channel that mediates the release of adenosine triphosphate (ATP)^15^, ^16^ and is involved in immune responses.^17^, ^18^ ATP can stimulate many receptors, including ligand‐gated cationic channels termed purinergic receptors type 2 X (P2X); there are seven mammalian P2X subtypes, namely, P2X1–7, that play various pathological roles.^19^ However, the exact role of PANX1‐ATP signalling in LPS‐induced endotoxemia remains controversial. In previous studies, inhibition of PANX1 has been shown to be beneficial for sepsis‐induced organ injury^20^, ^21^ and endotoxic shock.^22^ The mechanisms underlying the protective effect identified in these studies were partially elucidated by evidence showing decreased levels of proinflammatory cytokines, including interleukin (IL)‐1β, IL‐18 and tumour necrosis factor (TNF)‐α.^20^, ^21^, ^23^ In contrast to the results published by Yang et al.,^22^ Zhang et al. showed that inhibition of PANX1 does not significantly prolong survival after sepsis.^23^ Furthermore, as shown in the study by Chen et al., ^10^Panx, an inhibitor of PANX1, exacerbates sepsis‐induced death in mice, reducing survival rates from 60%–70% to 0%–10%; these results indicated an effect opposite to those observed in other studies.^24^ In another study by Li et al., Panx1 knockout impaired host immune defences and survival but reduced tissue damage after sepsis.^25^ Consequently, the specific roles of PANX1 in sepsis and the related organ injury remain largely unclear.

PANX1 plays a key role in the immune system through ATP‐driven activation of the NACHT, LRR and PYD domains‐containing protein 3 (NLRP3) inflammasome.^26^ Inflammasome activation results in the assembly of scaffold components, the cytoplasmic receptor NLRP3, the adaptor protein ASC, and the effector protein caspase‐1.^27^ Caspase‐1 induces the processing and release of IL‐1β and IL‐18.^28^ The full‐length functional IL‐33 protein, which is another IL‐1 family cytokine, has a molecular weight of 32 kDa but is not activated by caspase‐1‐mediated cleavage.^29^, ^30^ Crosstalk between PANX1 and IL‐33 has occasionally been reported, and the underlying mechanisms remain unclear. IL‐33 is a tissue‐derived nuclear cytokine whose expression by hepatocytes is induced during acute inflammation.^31^, ^32^ IL‐33 is involved in host defence, tissue repair and homeostasis and functions by targeting macrophages, regulatory T cells (Tregs) and other immune cells that express its receptor, IL1RL1 (ST2).^31^, ^33^ In contrast to recruiting macrophages that protect against MRSA,^9^ we demonstrated that IL‐33 increased the number of liver‐infiltrating ST2^+^ Tregs and promoted the resolution of hyperinflammation in LPS‐induced endotoxemia.

In this study, we found that the PANX1‐IL‐33 axis played a protective role against LPS‐induced endotoxemia by promoting the resolution of hyperinflammation. We initially observed low expression levels of proteins involved in the PANX1–IL‐33 axis in clinical patients with sepsis after LT. Next, we studied the landscape of immune responses after LPS challenge in Panx1‐deficient (Panx1^−/−^) mice. Furthermore, an orthoptic mouse LT model was established to study the liver‐specific role of the PANX1–IL‐33 axis in LPS‐induced endotoxemia. Based on the *in vitro* and *in vivo* results, we demonstrated that P2X7, but not P2X2, regulated PANX1‐dependent IL‐33 induction after LPS administration. Finally, rIL‐33 administration rescued LPS‐induced endotoxemia by increasing the numbers of liver‐infiltrating ST2^+^ Tregs and promoting the resolution of excessive inflammation. Here, we studied a novel mechanism by which the hepatic PANX1–IL‐33 axis is regulated by the ATP‐P2X7 pathway, targeting adaptive immunity in LPS‐induced endotoxemia; these findings supplement our previous study and others.^9^, ^34^

## 2 METHODS AND METHODS

### 2.1 Patients

Donor graft tissue specimens were harvested during liver graft procurement prior to LT at Shanghai General Hospital between January 2013 and December 2018. The liver tissue specimens were stored at –80°C. Proteins were extracted from the donor liver specimens after the recipients were divided into the sepsis group or control group. Patients who developed sepsis one week to six months after LT were enrolled in this study to eliminate the potential influence of ischemia‐reperfusion. All the patients with a liver injury who were enrolled in this study underwent a biopsy to eliminate the possibility of rejection. All the data are representative of at least three independent *in vitro* experiments performed in triplicate or at least two independent *in vivo* experiments.

### 2.2 Definition of sepsis

Sepsis is defined as life‐threatening organ dysfunction caused by a dysregulated host response to infection. Patients who underwent LT and had a sequential organ failure assessment score of 2 or more were diagnosed with sepsis.^3^

### 2.3 Animals and treatment protocols

Wild‐type (WT) C57BL/6 mice were obtained from the Shanghai Laboratory Animal Center (Shanghai, China). Panx1‐knockout mice were littermates that were used in our previous study.^9^ All the animals received humane care according to the criteria outlined in the “Guide for the Care and Use of Laboratory Animals” prepared by the National Academy of Sciences and published by the National Institutes of Health (NIH publication 86‐23 revised 1985). The mice were housed in the animal facility of the Institut Pasteur of Shanghai and received standard animal management. To eliminate potential differences caused by sample selection, all the experiments were conducted with 6‐ to 8‐week‐old male mice. Liver injury was established by an intraperitoneal injection of LPS (*Escherichia coli* 0111:B5; Sigma–Aldrich, St. Louis, MO, USA) dissolved in phosphate‐buffered saline (PBS) at doses of 10, 15, or 30 mg/kg body weight. Adeno‐associated viruses (AAVs) encoding short hairpin RNA (shRNA) targeting P2x7 or Panx1 (GenePharma, Shanghai, China) were injected via the hydrodynamic tail vein. Some mice were pretreated with a single intraperitoneal injection of PBS vehicle or rIL‐33 (2 μg/mouse) (R&D Systems, Minneapolis, MN, USA) 12 h prior to LPS administration.

### 2.4 Orthotopic LT model

WT or Panx1^−/−^ C57BL/6 mice were used as donors and recipients, respectively. A total of 4 groups of mice that underwent liver transplant were established: WT mice transplanted with WT (WT → WT) or Panx1^−/−^ liver allografts (Panx1^−/−^ → WT) and Panx1^−/−^ mice transplanted with WT (WT → Panx1^−/−^) or Panx1^−/−^ liver allografts (Panx1^−/−^ → Panx1^−/−^). LT surgery was performed as previously described.^9^ In brief, the liver was harvested and preserved in 4°C saline, and then, the donor's liver was transplanted into the recipient mice with the cuff anastomosis method (Figure S5). The mice that received liver transplants were treated with LPS 2 weeks after surgery when the LT model was stable.

### 2.5 Primary hepatocyte isolation

Primary murine hepatocytes were isolated by collagenase IV (Roche, Basel, Switzerland) perfusion and Percoll (GE Healthcare, Little Chalfont, Buckinghamshire, UK) gradient purification. In brief, each mouse was dissected after being anaesthetized to expose the portal vein, abdominal aorta and inferior vena cava. After ligation of the abdominal aorta, a trocar was inserted into the inferior vena cava. Then, the portal vein was cut, and perfusion and digestion were performed. Primary murine hepatocytes were seeded at 2 × 10^5^ cells/well in collagen Ⅰ (Sigma–Aldrich)‐coated 6‐well plates and cultured in Dulbecco's modified Eagle's medium (DMEM) (Gibco BRL, Grand Island, NY, USA).

### 2.6 Cell line and treatment

An immortalized human liver cell line (L02) was obtained from the Shanghai Institute for Biological Sciences, Chinese Academy of Sciences (Shanghai, China). L02 cells were cultured in DMEM supplemented with 10% fetal bovine serum (FBS) (Gibco BRL) and 1% penicillin/streptomycin (HyClone, South Logan, UT, USA). L02 cells retain the typical features of normal primary hepatocytes, including the cell morphology and the production of albumin, during culture. L02 cells were stimulated with 1 μg/ml LPS (Sigma–Aldrich) dissolved in PBS or vehicle for various periods. PANX1 channel inhibitors and scrambled control (^10^Panx, scrambled ^10^Panx: APExBIO, Houston, TX, USA; Probenecid: Sigma–Aldrich) and inhibitors of P2Xs (NF‐449, RO‐3, NF‐110, and 5‐BDBD: Tocris, Bristol, UK; KN‐62: MedChem Express, Monmouth Junction, NJ, USA) were administered 30 min prior to stimulation. ATP (Sigma–Aldrich) was added at concentrations ranging from 0.1 to 5 mM 45 min prior to sample collection.

### 2.7 Quantitative analysis of cytokines and chemokines

Mouse serum levels of IFN‐γ, IL‐12p70, IL‐13, IL‐1β, IL‐2, IL‐4, IL‐5, IL‐6, IL‐10, IL‐18, IL‐33, TNF‐α, GM‐CSF, GRO‐α, IP‐10, MCP‐1, MCP‐3, MIP‐1α, MIP‐1β, MIP‐2, and RANTES were measured with Luminex kits (eBioscience, San Diego, CA, USA) according to the manufacturer's instructions. Several independent IL‐33 levels were measured by enzyme‐linked immunosorbent assay (ELISA) (Westang Biotech, Shanghai, China) according to the manufacturer's protocol.

### 2.8 Immunohistochemistry and immunofluorescence staining

Mouse tissues were fixed using 4% formaldehyde and then embedded in paraffin. After deparaffinization and rehydration, 5‐μm‐thick sections were stained with hematoxylin and eosin (HE) or primary antibodies (anti‐IL‐33, anti‐cleaved caspase‐3 (cl.Casp3), anti‐caspase‐8, and anti‐caspase‐9 antibodies: Cell Signaling Technology, Danvers, MA, USA), followed by incubation with horseradish peroxidase (HRP)‐conjugated secondary antibody (Abcam, Cambridge, MA, USA) and processing with a DAB substrate kit (Abcam). The cells that were stained brown were considered positive.

Terminal deoxynucleotidyl transferase‐mediated dUTP nick end labelling (TUNEL) staining was performed with a TUNEL assay kit (Roche) after antigen retrieval and permeabilization. Double‐strand breaks were labelled with FITC. The nucleus was labelled with DAPI (Sigma–Aldrich) and appeared blue. All the slides were visualized using immunofluorescence microscopy (Olympus, Tokyo, Japan).

### 2.9 Western blotting

Liver specimens or cell cultures were lysed in RIPA buffer (Thermo Fisher Scientific), supplemented with protease inhibitors (Roche) for 30 min at 4°C, and then, the samples were centrifuged at 13,000 rpm at 4°C for 10 min. The protein concentrations in the supernatants were measured with the Bradford method (Bio‐Rad, Hercules, CA, USA). Then, the proteins were mixed with SDS sample buffer and boiled for 5 min. After separation by SDS–PAGE, the proteins were transferred to nitrocellulose membranes, which were blocked with nonfat milk and incubated with primary antibodies (anti‐β‐actin antibody, anti‐caspase‐1 antibody and anti‐IL‐33 antibody: Abcam; anti‐NLRP3 antibody, anti‐P2X2 antibody, anti‐P2X4 antibody, anti‐P2X7 antibody, anti‐IL‐1β antibody, and anti‐IL‐18 antibody: Cell Signaling Technology) at 4°C overnight. The membranes were washed with TBST (20 mM Tris‐HCl, 150 mM NaCl, and 1% Tween‐20) and incubated with HRP‐conjugated secondary antibodies (1:5,000) (Abcam) for 1 h. Protein‐antibody complexes were visualized using enhanced chemiluminescence (Merck Millipore, Billerica, MA, USA), and the blots were exposed to X‐ray films. The expression of each protein was quantified as the ratio of the band intensity of each protein to the band intensity of β‐actin using Photoshop software (Adobe Systems, Incorporated, San Jose, CA, USA).

### 2.10 Quantitative real‐time PCR

Total RNA was extracted from mouse liver tissue with TRIzol (Invitrogen, Carlsbad, CA, USA). cDNA synthesis was performed with Superscript III reverse‐transcription reagent (Invitrogen) using 1 μg RNA. Quantitative real‐time PCR was conducted with SYBR Green I dye (Roche, Basel, Switzerland). PCR cycling started at 95°C for 30 seconds followed by 40 cycles of 95°C for 5 seconds and 60°C for 30 seconds, with the last step at 72°C for 20 min. The primer sequences used were listed in our previous study.^9^ Relative mRNA level changes were analyzed using the 2^−ΔΔCt^ method.

### 2.11 Flow cytometry

For surface marker staining, cells were stained in PBS supplemented with 1% FBS for 30 min on ice with the following antibodies (PerCP‐conjugated anti‐CD3, BV421‐conjugated anti‐CD4, PE‐conjugated anti‐CD8, BV570‐conjugated anti‐CD45, BV421‐conjugated anti‐F4/80, BV650‐conjugated anti‐CD86, APC‐conjugated anti‐CD206, PE‐Cy7‐conjugated anti‐CD25, FITC‐conjugated anti‐γδ TCR, APC‐Cy7‐conjugated anti‐CD11b, PE‐conjugated anti‐Ly‐6G, FITC‐conjugated anti‐CD49b, PE‐conjugated anti‐CD19, PE‐conjugated anti‐ST2 antibodies, and APC‐conjugated anti‐NKp46: BD Biosciences, San Jose, CA, USA). After fixation and permeabilization, staining with an APC‐conjugated anti‐FOXP3 antibody (eBioscience) was performed according to the manufacturer's instructions. For IL‐33 staining of primary mouse hepatocytes, cells were stained using a biotinylated anti‐IL‐33 monoclonal antibody (Enzo Life Biosciences, Raamsdonksveer, The Netherlands) and PE‐Cy7‐labeled streptavidin (BD Pharmingen, San Diego, CA, USA). All the flow cytometric data were analyzed and plotted using FlowJo software (TreeStar, Ashland, OR, USA).

### 2.12 ATP measurements

Liver tissue interstitial fluid samples were collected following the method described in a previous study.^35^ ATP concentrations were measured using the ATP Determination Kit (Beyotime Biotechnology, Shanghai, China) and a luminometer according to the manufacturer's instructions.

### 2.13 Statistics

Continuous data are expressed as the mean and standard deviation, while discrete variables are shown as frequencies. Categorical variables were compared using Pearson's χ2 test or Fisher's exact test, whereas continuous variables were assessed using Student's *t*‐test, the Mann–Whitney U test or a one‐way analysis of variance. Survival rates were assessed using Kaplan–Meier analysis, and differences between subgroups were compared using the log‐rank test. All the statistical analyses were performed with GraphPad Prism 5 (GraphPad Software, La Jolla, CA, USA). Differences were considered statistically significant when P < 0.05. Significance is shown as follows: ^*^ *p* < 0.05, ^**^ *p* < 0.01, and ^***^ *p* < 0.001.

### 2.14 Study approval

No donor organs were obtained from executed prisoners or other institutionalized persons. Written informed consent was received from donors and recipients prior to inclusion in the study. This study was approved by the Institutional Review Board of Shanghai General Hospital, Shanghai Jiao Tong University School of Medicine (2015KY073) and was conducted in accordance with the 1975 Declaration of Helsinki and its later revisions.^36^ The experimental procedures and animal use and care protocols were approved by the Institutional Animal Care and Use Committee at the Institut Pasteur of Shanghai.

### 2.15 Data availability

The data that support the findings of this study are available from the corresponding author upon reasonable request.

## 3 RESULTS

### 3.1 The PANX1–IL‐33 axis was associated with sepsis and liver injury

**FIGURE 1:**
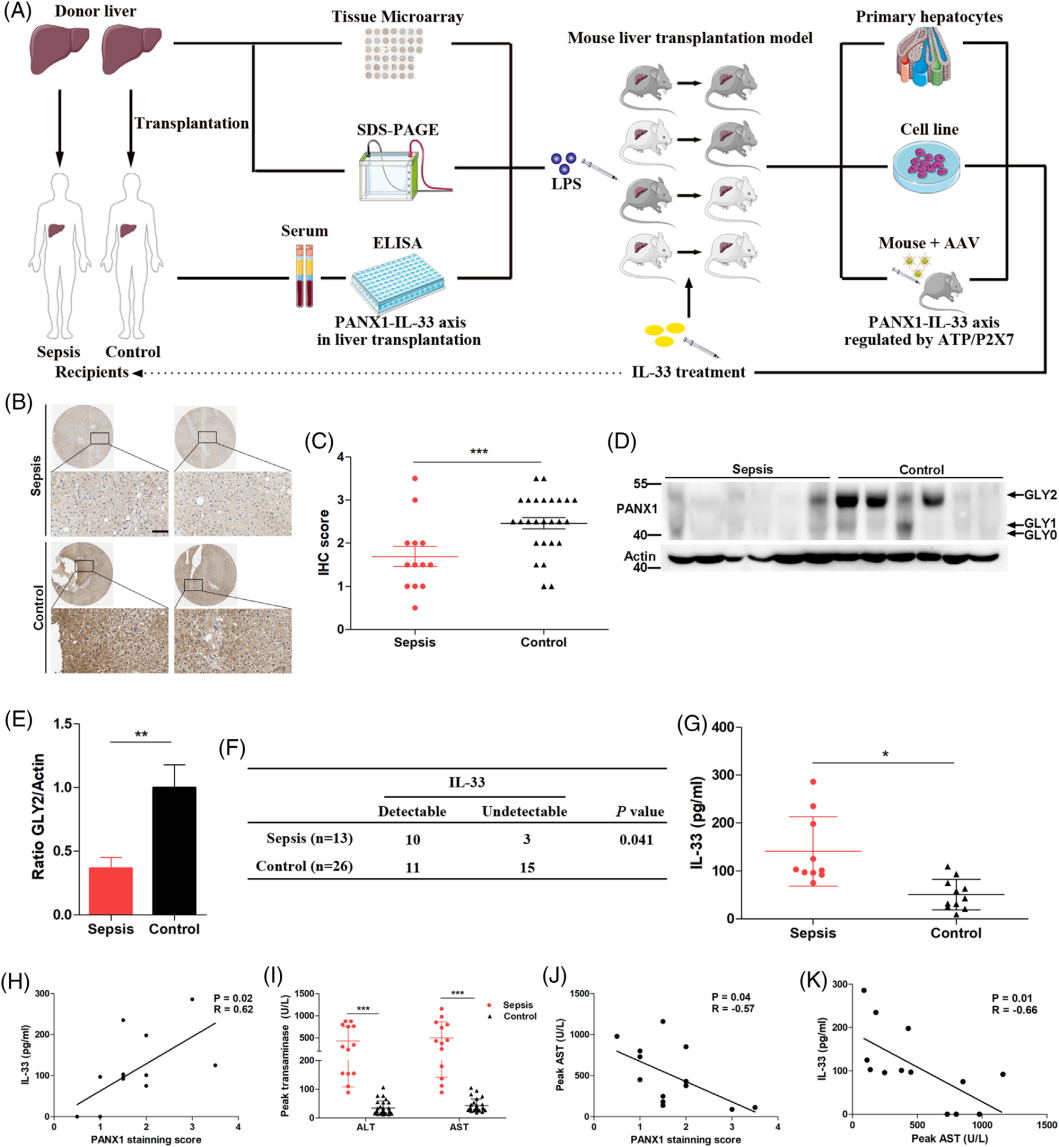
The PANX1–IL‐33 axis was associated with sepsis in liver transplantation (LT) patients. (A) Experimental design of this study. (B and C) Representative images and quantification of immunohistochemistry (IHC) staining for PANX1, which was evaluated blindly by two pathologists, in an independent cohort comprising 13 donor grafts of septic recipients and 26 grafts of uninfected controls that were obtained during liver graft procurement (scale bar = 50 μm). (D and E) Immunoblot and relative quantification of PANX1 GLY2 in donor graft tissue samples from patients with sepsis (*n* = 6) and control subjects (*n* = 6) prior to liver transplantation (LT). (F and G) Serum IL‐33 levels measured one week after diagnosis in the sepsis group and at matched time points in the control group. (H) Correlation between circulating IL‐33 levels after LT and the PANX1 staining score of donor graft tissue samples prior to LT (*n* = 13). (I) Peak alanine aminotransferase (ALT) and aspartate aminotransferase (AST) levels in patients with sepsis (*n* = 13) compared with control subjects (*n* = 26). (J) Correlation between the peak AST levels measured during sepsis after LT and the PANX1 staining score of donor graft tissue samples prior to LT (n = 13). (K) Correlation between circulating IL‐33 levels after LT and peak AST levels measured during sepsis after LT (*n* = 13). *p < 0.05 and ***p < 0.001 by Student's t‐test or one‐way analysis of variance (ANOVA). Correlations were analyzed using Pearson's correlation test

To study whether PANX1–IL‐33 signalling plays a role in the defence against sepsis caused by GNB infection,^9^ we analyzed the PANX1–IL‐33 axis in a cohort comprising 13 patients with sepsis caused by GNB and 26 patients with no infection; this cohort is independent of those examined in our previous studies^9^, ^37^ (Figure 1A). The analyzed donor graft tissue samples were obtained during liver graft procurement prior to LT. The donor and recipient information is summarized in Table 1. The baseline characteristics were comparable between the two groups.

**TABLE 1:** Donor and recipient characteristics

	No infection (*n* = 26)	Sepsis (*n* = 13)	*p*
Donor age, years	42 (26–47)	45 (28–49)	0.72
Donor sex (male/female)	20/6	9/4	0.70
Donor BMI, kg/m^2^	22.8 (20.9–25.1)	22.8 (21.9–25.4)	0.63
Donor cause of death			0.58
Anoxia	2 (7.7%)	2 (15.4%)	
Brain tumor	3 (11.5%)	0 (0%)	
Trauma	14 (53.8%)	6 (46.2%)	
Stroke	7 (26.9%)	5 (38.5%)	
Cold ischemia time, minutes	267 (219–313)	281 (234–321)	0.26
Recipient age, years	46 (40–51)	47 (42–50)	0.82
Recipient, sex (male/female)	21/5	9/4	0.45
Recipient BMI, kg/m^2^	23.7 (20.8–25.2)	22.3 (21.1–25.4)	0.42
Recipient primary disease			0.91
HBV	19 (73.1%)	11 (84.6%)	
Acute liver failure	2 (7.7%)	0 (0%)	
Alcoholic cirrhosis	2 (7.7%)	1 (7.7%)	
Other	3 (11.5%)	1 (7.7%)	
MELD score > 30	1 (3.8%)	2 (15.4%)	0.25
Corticosteroids or ATG therapy, no (%)	3 (11.5%)	3 (23.1%)	0.35
Ventilation, hours	13 (8–33)	17 (14–53)	0.11
Biliary complications post‐LT, no (%)	1 (3.8%)	2 (15.4%)	0.25
Reoperation, no (%)	1 (3.8%)	1 (7.7%)	>0.99

Abbreviations: ATG, anti‐thymocyte globulin. Continuous data are presented as medians with interquartile ranges; BMI: body mass index; categorical data are presented as counts and percentages.; HBV: hepatitis B virus; HCC: hepatocellular carcinoma; MELD, model for end‐stage liver disease.

The 39 slides were immunohistochemically stained for PANX1 expression and analyzed. Representative images are shown in Figure 1B, and quantitative analysis of the immunohistochemical staining revealed a significantly lower level of PANX1 in the donor graft tissue samples from patients with sepsis than in the samples from control subjects (Figure 1C). PANX1 is present in three glycosylation states—a nonglycosylated protein (GLY0), a high‐mannose glycoprotein (GLY1), and a fully mature glycoprotein (GLY2).^38^ GLY2 represents the mature and main part of the membrane‐bound PANX1 channel protein according to our Western blotting analysis of the donor graft tissue samples (Figure 1D). The results showed significantly decreased GLY2 levels in the donor graft samples from patients with sepsis (Figure 1E).

Next, we measured circulating IL‐33 levels in these 39 patients. Sera were collected from 13 septic recipients 1 week after sepsis onset, and sera were harvested from 26 control patients at a matched timepoint and at a ratio of 2:1. We found that circulating IL‐33 levels were detectable in significantly more patients in the sepsis group (77%, 10/13) than in the control group (42%, 11/26) (Figure 1F), and the serum IL‐33 levels in other patients were below the limit of detection of the enzyme‐linked immunosorbent assay (ELISA). In the patients with detectable IL‐33 levels, those in the sepsis group showed a significantly higher circulating IL‐33 level than those in the control group (Figure 1G). Consistent with a previous study, sepsis increased circulating IL‐33 levels.^39^ Since IL‐33 levels were not increased in the control group, we analyzed the 13 patients with sepsis who had increased circulating IL‐33 levels. A significant positive correlation between the PANX1 immunohistochemical staining score and circulating IL‐33 levels was observed (Figure 1H).

The peak alanine transaminase (ALT) and aspartate aminotransferase (AST) levels in the recipients with sepsis were measured during the infection, and the levels were significantly higher than in recipients without sepsis in controls (Figure 1I); these results suggested that sepsis caused damage to the transplanted liver. Furthermore, we analyzed the correlation between PANX1 levels and peak AST levels in 13 patients with sepsis to further study the role of PANX1 in sepsis‐induced liver injury. As shown in Figure 1J, the peak AST level during sepsis was negatively correlated with donor PANX1 expression prior to LT. Similarly, the circulating IL‐33 levels were also negatively correlated with the peak serum AST levels during sepsis (Figure 1K). Collectively, we demonstrated the vital role of the PANX1–IL‐33 axis in sepsis and related liver injury using clinical samples.

**FIGURE 2:**
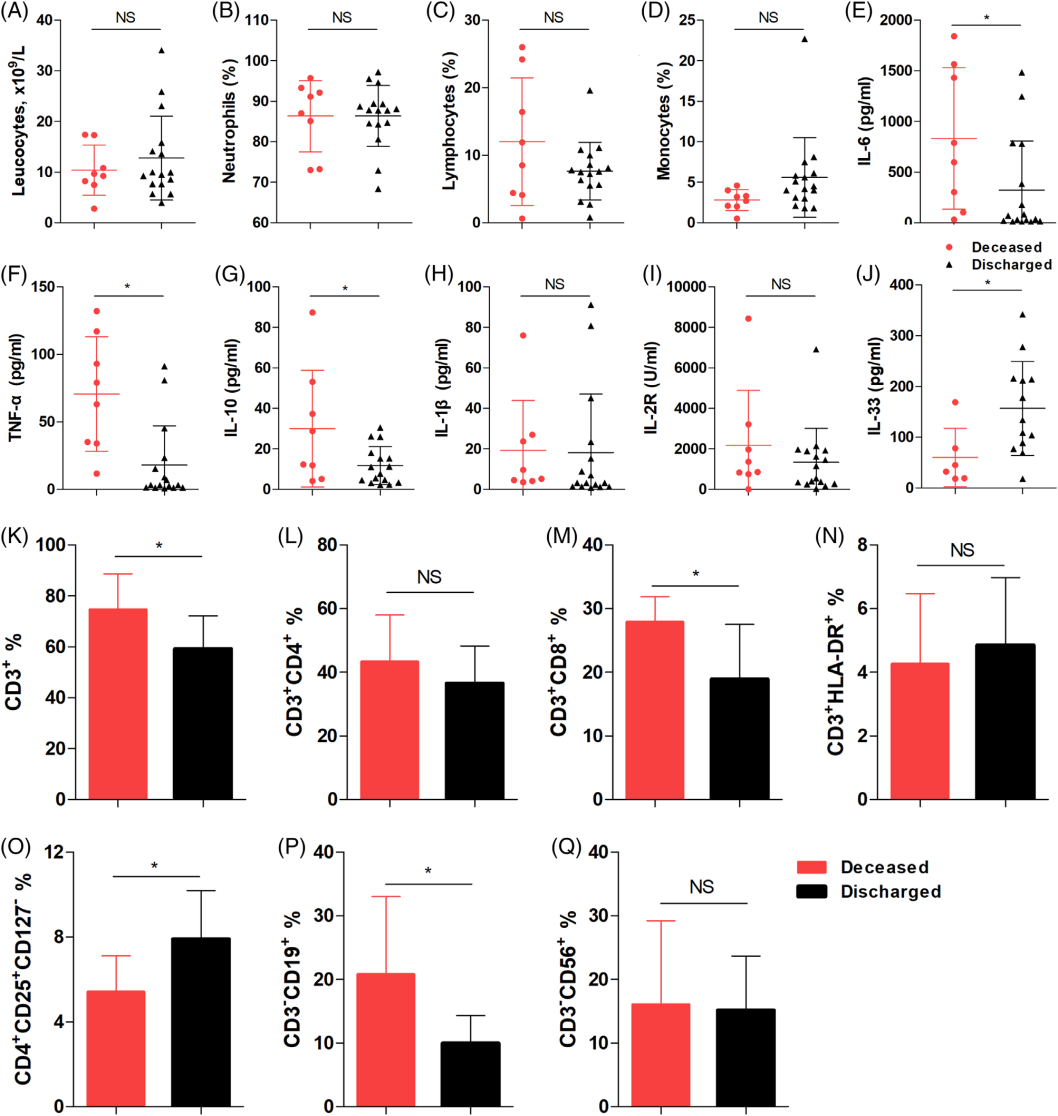
Immune cell and cytokine levels in septic patients after liver transplantation (LT). (A) Blood leukocyte numbers and proportions of (B) neutrophils, (C) lymphocytes, and (D) monocytes from septic patients in the deceased group (*n* = 8) and the discharged group (*n* = 16). Serum levels of (E) IL‐6, (F) TNF‐α, (G) IL‐10, (H) IL‐1β, (I) IL‐2R and (J) IL‐33 in septic patients in the deceased group (n = 8) and discharged group (*n* = 16). Proportions of (K) CD3^+^ T cells, (L) CD3^+^CD4^+^ T cells, (M) CD3^+^CD8^+^ T cells, (N) CD3^+^HLA‐DR^+^ T cells, (O) CD4^+^CD25^+^CD127^−^ regulatory T cells, (P) CD3^−^CD19^+^ B cells, and (Q) CD3^−^CD56^+^ NK cells in the blood of septic patients in the deceased group (*n* = 8) and discharged group (*n* = 16). *p < 0.05 by Student's t‐test

To study the immune landscape of septic patients after LT, we analyzed the cytokine and immune cell levels during infection in another independent cohort with sepsis caused by GNB infection (Figure 2). A total of 24 septic patients were analyzed, with8 patients in the deceased group and 16 in the discharged group. These data were recorded at the time point when circulating IL‐6 levels were the highest during sepsis. As shown in Figure S2A–D, there were no significant differences in leucocyte, neutrophil, lymphocyte or monocyte numbers between the two groups. The IL‐6, TNF‐α and IL‐10 levels were significantly higher (Figure 2E–G) while the IL‐33 levels were significantly lower (Figure 2J) in the deceased group than in the discharged group. Furthermore, the patients in the deceased group had significantly higher proportions of CD3^+^ T cells (Figure 2K), CD3^+^CD8^+^ T cells (Figure 2M), and CD3^−^CD19^+^ B cells (Figure 2P) and lower proportions of CD4^+^CD25^+^CD127^−^ regulatory T cells (Figure 2O) in their blood. Other detected cytokine (Figure 2H,I) and immune cell (Figure 2L,N,Q) levels were comparable between the two groups. These data suggest that deceased septic patients had more severe dysregulated immune responses and lower IL‐33/Treg levels, which suggested that the uncontrolled hyperinflammation state was associated with a worse prognosis in sepsis patients.

### 3.2 PANX1 deficiency increased the severity of LPS‐induced endotoxemia and liver injury in mice

**FIGURE 3:**
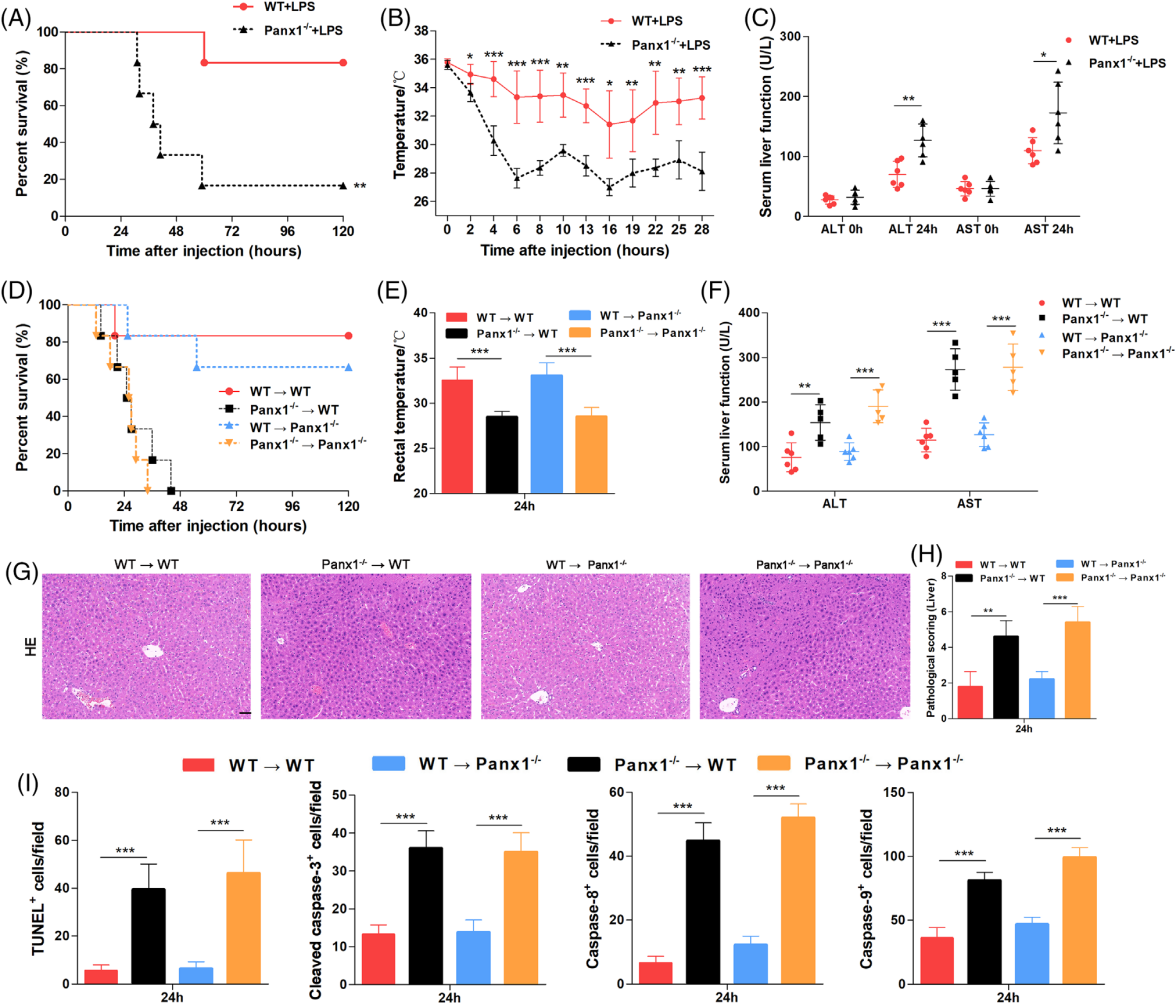
Protective role of hepatic PANX1 in endotoxemia and the liver *in vivo*. (A) Survival (*n* = 6 mice per group), (B) rectal temperatures (*n* = 5 mice per group), and (C) serum ALT and AST levels (*n* = 5 mice per group) of Panx1^−/‐^ and WT mice after injection with 15 mg/kg lipopolysaccharide (LPS). (D) Survival (*n* = 6 mice per group), (E) rectal temperatures, and (F) serum ALT and AST levels of WT → WT, Panx1^−/−^ → wild‐type (WT), WT → Panx1^−/−^, and Panx1^−/−^ → Panx1^−/−^ mice at 24 h after injection with 15 mg/kg LPS. (G and H) Representative images and quantification of hematoxylin and eosin staining and (I) quantification of TUNEL staining and IHC staining for cleaved caspase‐3, caspase‐8 and caspase‐9 in liver tissue samples from the four groups of mice 24 h after injection with 15 mg/kg LPS (scale bar = 50 μm). The quantified results are the average of 10 images per mouse (*n* = 5 mice per group). *p < 0.05, **p < 0.01, and ***p < 0.001 by Student's t‐test, one‐way ANOVA, or log‐rank test

Considering that LPS plays a central role in clinical patients with sepsis caused by GNB infection, we established LPS‐induced endotoxemia in mice. According to previous studies, the exact role of PANX1 in endotoxemia remains controversial.^20^, ^21^, ^22^, ^23^, ^24^, ^25^ Therefore, to directly examine the role of PANX1 in endotoxemia and liver injury, we utilized Panx1‐deficient (Panx1^−/−^) mice. The Panx1^−/‐^ mice used in this study were viable, appeared normal and healthy, and showed no significant phenotypes. We injected different doses of LPS into Panx1^−/‐^ mice and wild‐type (WT) mice. The mortality in the Panx1^−/−^ mice was significantly higher than that in the WT mice after administration of 15 mg/kg LPS (Figure 3A). Similarly, when LPS was administered at a dose of 30 mg/kg, the Panx1^−/−^ mice were also more susceptible to LPS challenge than the WT mice (Figure S1A). Since there was a more significant difference when a dose of 15 mg/kg LPS was administered, we performed subsequent experiments under this condition. Consistent with this finding, the rectal temperature of the Panx1^−/−^ mice at various time points robustly decreased compared to that of the WT mice (Figure 3B). We observed that the Panx1^−/−^ mice had more severe clinical symptoms of LPS‐induced shock, including hypothermia, shivering and low reactivity, than the WT mice. Together, these results suggest that PANX1 plays a vital role in LPS‐induced systemic endotoxic shock.

The liver receives a high level of LPS through the portal vein and/or systemic circulation due to the gut‐liver axis. Next, we investigated whether PANX1 protects the liver from LPS‐induced injury. Panx1^−/−^ mice exhibited significantly increased serum levels of alanine, ALT and AST after the LPS challenge (Figure 3C). Liver sections harvested from the Panx1^−/−^ mice at 24 h after LPS injection showed more severe liver damage than those from the WT mice, as demonstrated by HE staining (Figure S1B and C). Liver piecemeal necrosis was observed in the Panx1^−/−^ mice, while spotty necrosis was more commonly observed in the WT mice. In addition, immunohistochemistry (IHC) for cleaved caspase‐3 (Figure S1D,E) and terminal deoxynucleotidyl transferase‐mediated dUTP nick end labelling (TUNEL) (Figure S1F,G) both showed increased numbers of apoptotic cells in the livers of Panx1^−/−^ mice. Thus, PANX1 exerted a protective effect against LPS‐induced endotoxic shock and the related liver injury.

### 3.3 Deficiency of PANX1 induced dysregulated immune responses in mice

Endotoxemia is characterized by uncontrolled hyperinflammation and dysregulated immune responses. To study the immune landscape of Panx1^−/−^ mice with endotoxemia, we measured the levels of cytokines, chemokines and various immune cells in the host circulation.

We demonstrated that the administration of LPS resulted in significantly higher serum IL‐6, TNFα, IFN‐γ, IL‐2, IL‐4, IL‐13, IL‐18, and IL‐1β levels in the Panx1^−/‐^ mice than in the WT mice at 24 h (Figure S2A–H). Interestingly, the serum levels of TNF‐α, IL‐18, and IL‐1β (Figure S2B,G,H) in the Panx1^−/‐^ mice tended to be lower at 12 h than those in the WT mice; however, the differences were not significant. Notably, the situation was reversed at 24 h, which was consistent with the more severe inflammatory cascade in Panx1^−/‐^ mice. In contrast, the serum IL‐33 levels were significantly decreased at 12 h and remained lower at 24 h in the Panx1^−/‐^ mice than in the WT mice (Figure S2I). The levels of the other cytokines and chemokines, including IL‐10, IL‐12p70, GM‐CSF, CXCL10, RANTES, IL‐5, MCP‐1, MIP‐2, GRO‐α, MIP‐1α, and MCP‐3, are shown in Figure S3A–K. The levels of most of these proteins remained substantially increased in the Panx1^−/‐^ mice at 24 h, while the levels in the WT mice returned to basal levels by this time point. Therefore, the loss of PANX1 led to uncontrolled hyperinflammation during endotoxemia. The cytokine storm was a critical reason why the Panx1^−/‐^ mice became moribund and began to die at approximately 24 h.

Because of the lethality of 15 mg/kg LPS, we investigated the effect of Panx1 deficiency on immune cell populations in the mice 3 days after injection with 10 mg/kg LPS. We observed significantly fewer CD4^+^CD8^+^ double‐positive (DP) thymocytes in the thymus of the Panx1^−/‐^ mice than in the WT mice after LPS stimulation (Figure S2J,K), suggesting that Panx1 deficiency leads to more serious thymic involution in a mouse model of endotoxemia.^40^ Among the lymphocytes examined in the lymph nodes, significantly more CD19^+^ B cells (Figure S2L,M) and fewer Tregs (Figure S2N,O) were observed in the lymph nodes of the Panx1^−/‐^ mice compared with those of the WT mice after LPS stimulation. The levels of some other cell subtypes, including CD3^+^CD4^+^ T cells, CD3^+^CD8^+^ T cells, γδ T cells, macrophages, natural killer T cells and neutrophils, are shown in Figure S3L–V and these levels were comparable between the Panx1^−/‐^ and WT mice. In addition to the number of macrophages, the polarization of macrophages was also of great significance for understanding the immune status of the Panx1^−/−^ mice after LPS administration. Classically activated macrophages (M1 macrophages) and alternatively activated macrophages (M2 macrophages) perform distinct functions in various diseases. In terms of inflammation, M1 macrophages are considered proinflammatory, while M2 macrophages are considered anti‐inflammatory. We therefore further studied the proportions of M1‐like macrophages and M2‐like macrophages in the liver (Figure S4A–C), blood (Figure S4D–F), spleen (Figure S4G–I) and lymph nodes (Figure S4J–L) from the Panx1^−/−^ mice and WT mice after LPS injection. The proportions of M1‐like macrophages and M2‐like macrophages markedly changed after LPS injection in both the Panx1^−/−^ mice and WT mice (Figure S5). However, there was no significant difference between the Panx1^−/−^ mice and WT mice. These data together suggested that Panx1 deficiency‐induced systemic hyperinflammation delayed its resolution in LPS‐induced endotoxemia.

### 3.4 Protective effect on endotoxemia and relative liver injury in a mouse LT model

To study the role of hepatic PANX1 in endotoxemia and fully simulate clinical practice, we generated an orthoptic mouse LT model (Figure S5A) using WT mice or Panx1^−/−^ mice as donors and recipients, respectively. A total of four groups of LT mice were generated: WT mice transplanted with WT (WT → WT) or Panx1^−/−^ liver allografts (Panx1^−/−^ → WT) and Panx1^−/−^ mice transplanted with WT (WT → Panx1^−/−^) or Panx1^−/−^ liver allografts (Panx1^−/−^ → Panx1^−/−^) (Figure S5B). Consequently, we focused on the liver‐specific effects of PANX1 on hepatic resistance to endotoxemia.

Using the protocol described above, 15 mg/kg LPS significantly impaired survival of the Panx1^−/−^ → WT mice compared with the WT → WT mice (Figure 3D). Furthermore, the mice receiving Panx1^−/‐^ liver allografts showed significantly decreased rectal temperature compared with those receiving WT liver allografts (Figure 3E). Consistent with these findings, increased serum ALT and AST levels were observed in the mice receiving Panx1^−/‐^ liver allografts compared with those receiving WT liver allografts after the LPS challenge (Figure 3F). HE and TUNEL staining showed more serious liver injury in the mice receiving Panx1^−/‐^ liver allografts after LPS administration (Figure 3G–I; Figure S1H). In addition, more apoptotic hepatocytes were observed in the mice receiving Panx1^−/‐^ liver allografts after the LPS challenge, as shown by the staining of caspase family proteins (Figure 3I; Figure S1I–K). HE staining of the lungs and kidneys of the mice that received liver transplants was also performed (Figure S6A,B), and there was no significant difference in terms of lung and kidney injury at 24 h. Based on these findings, PANX1 in hepatocytes exerted a protective effect against endotoxemia and related liver injury.

### 3.5 Hepatic PANX1‐IL‐33 axis attenuated endotoxemia by targeting Tregs rather than macrophages

According to our results, the Panx1^−/‐^ mice exhibited a more severe cytokine storm (Figures S2 and S3) during endotoxemia and, in contrast, consistently decreased IL‐33 levels (Figure S2I). Furthermore, the numbers of Tregs, which could be amplified by IL‐33,^39^ were also decreased in the Panx1^−/‐^ mice (Figure S2N,O). To study the role of the liver‐specific effects of PANX1 on the cytokine storm and Treg numbers, we assessed these elements in a mouse LT model.

**FIGURE 4:**
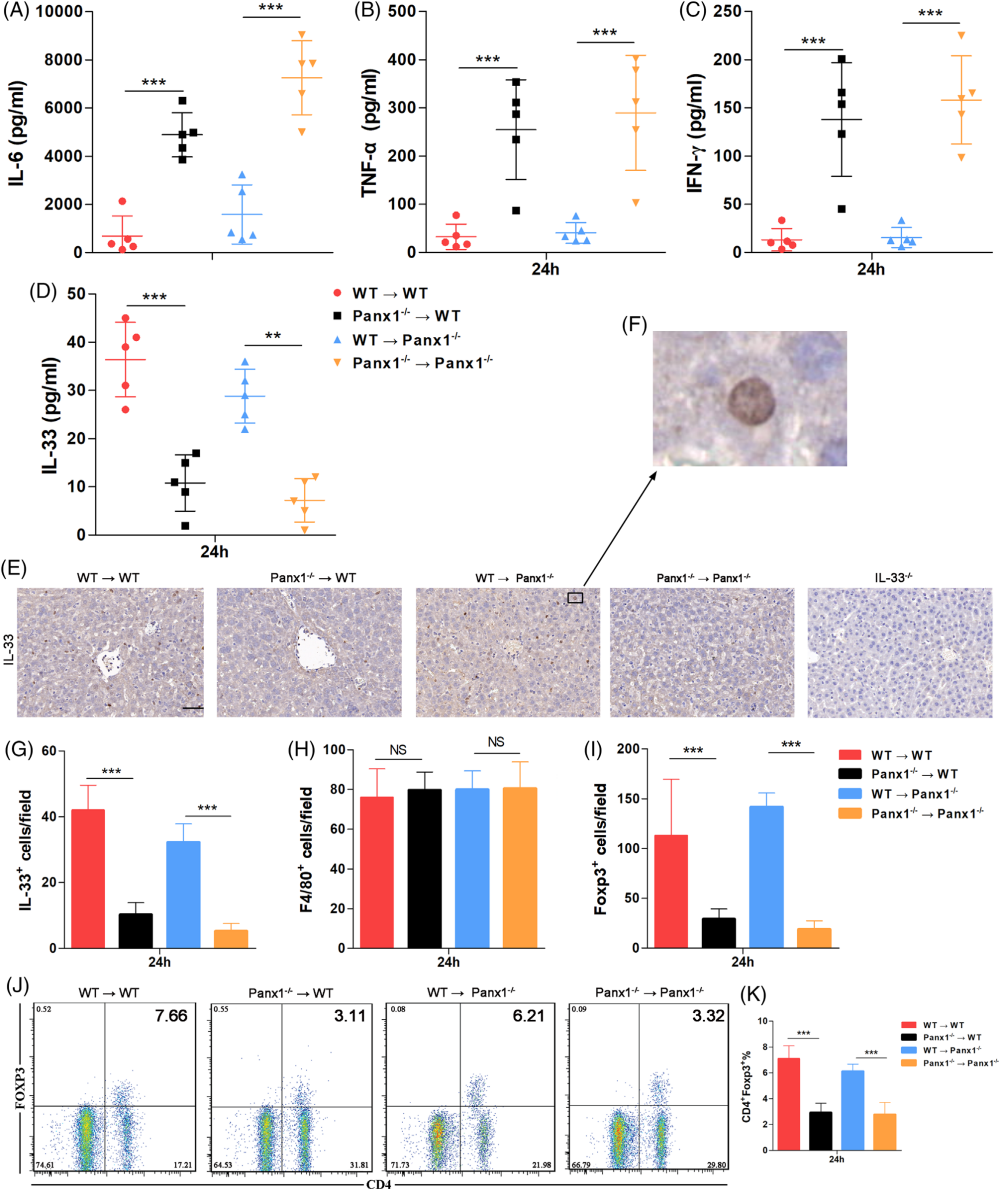
Hepatic PANX1‐IL‐33 axis deficiency results in dysregulated immune responses. Serum levels of (A) interleukin 6 (IL‐6), (B) tumour necrosis factor‐alpha (TNF‐α), (C) IFN‐γ, and (D) IL‐33 in wild‐type (WT) → WT, Panx1^−/−^ → WT, WT → Panx1^−/−^, and Panx1^−/−^ → Panx1^−/−^ mice at 24 h after injection with 15 mg/kg LPS (*n* = 5 mice per group). (E–G) Representative images and quantification of IHC staining for IL‐33 in the livers of the four groups at 24 h after injection with 15 mg/kg lipopolysaccharide (LPS). Staining of the livers from IL‐33^−/‐^ mice was used as a negative control. The quantified results are the average of 10 images per mouse (scale bar = 50 μm) (*n* = 5 mice per group). (H and I) Quantification of IHC staining for F4/80^+^ and Foxp3^+^ in the livers of the four groups at 24 h after injection with 15 mg/kg LPS. The quantified results are the average of 10 images per mouse (*n* = 5 mice per group). (J and K) Proportions of CD4^+^ Foxp3^+^ cells on CD3^+^ cells in the blood of mice from the four groups at 24 h after injection with 15 mg/kg LPS (*n* = 5 mice per group). *p < 0.05, **p < 0.01, and ***P < 0.001 by Student's t‐test or one‐way ANOVA

Using the protocol described above, as shown in Figure 4A–C, circulating IL‐6, TNF‐α, and IFN‐γ levels were significantly increased in the mice receiving Panx1^−/−^ liver allografts. In contrast, circulating IL‐33 levels were significantly decreased in the mice receiving Panx1^−/−^ liver allografts (Figure 4D). Next, we performed IHC staining of IL‐33 using mouse liver specimens to measure local IL‐33 expression in a mouse LT model. As shown in Figure 4E,F, IL‐33 was located in the nucleus of hepatocytes. Furthermore, the mice receiving Panx1^−/−^ liver allografts had significantly fewer IL‐33‐positive cells than those receiving WT liver allografts (Figure 4E,G).

Next, we assessed the macrophage and Treg populations in the liver, as these are reported to be two crucial groups of cells during organ damage. As shown in Figure 4H, F4/80 IHC staining of liver samples showed no significant differences among the four groups of the LT mouse model. In contrast, the mice receiving Panx1^−/−^ liver allografts presented significantly lower Treg numbers than those receiving the WT liver allografts (Figure 4I). Consistently, fewer circulating Tregs was observed in the mice receiving Panx1^−/−^ liver allografts (Figure 4J,K). These data suggested that the hepatic PANX1‐IL‐33 axis attenuates endotoxemia and related liver injury by targeting Tregs rather than macrophages.

### 3.6 PANX1 positively controlled IL‐33 synthesis in hepatocytes after LPS administration

**FIGURE 5:**
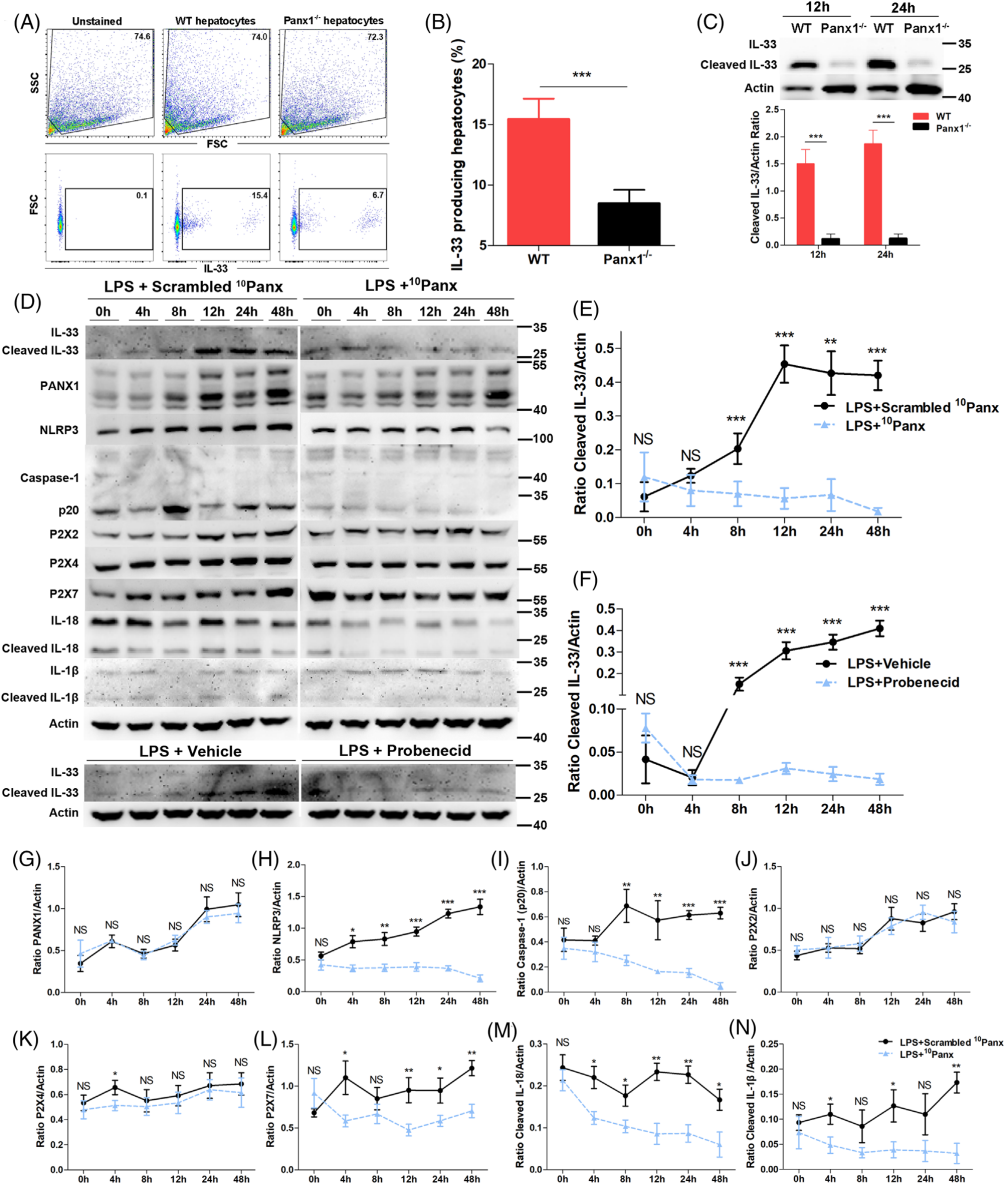
PANX1 positively regulates IL‐33 synthesis in hepatocytes after LPS administration. (A and B) Proportions of IL‐33^+^ primary mouse hepatocytes at 12 h after lipopolysaccharide (LPS) stimulation (1 μg/ml) (*n* = 5 per group). (C) Immunoblot and quantification of IL‐33 in primary WT hepatocytes and Panx1^−/−^ hepatocytes from mice at 12 and 24 h after LPS stimulation (1 μg/ml) (*n* = 4 per group). (D‐N) Immunoblots and quantification of the levels of IL‐33 and intermediates in the PANX1‐P2Xs‐NLRP3‐caspase‐1‐IL‐1 pathway in L02 cells treated with ^10^Panx (200 μM), scrambled ^10^Panx (200 μM), probenecid (50 μM) or vehicle at 0, 4, 8, 12, 24 and 48 h after LPS stimulation (1 μg/ml) (n = 4 per group). *p < 0.05, **p < 0.01 by Student's t‐test or ANOVA. Correlations were analyzed using Pearson's correlation test

Next, we examined whether PANX1 could regulate IL‐33 expression in hepatocytes after the LPS challenge. We performed *in vitro* experiments using both mouse primary hepatocytes and a human liver cell line to analyze the effect of PANX1 on IL‐33 expression. First, primary hepatocytes were isolated from WT and Panx1^−/‐^ mice to elucidate the effect of PANX1 on IL‐33 expression in hepatocytes at the genetic level. Primary hepatocytes were cultured for 2 days and then stimulated with LPS. We performed a flow cytometric analysis with intracellular IL‐33 staining. As shown in Figure 5A,B, significantly fewer IL‐33^+^ hepatocytes were observed among the Panx1^−/‐^ primary hepatocytes. The western blotting analysis also showed substantially decreased cleaved‐IL‐33 synthesis in the Panx1^−/−^ hepatocytes at both 12 and 24 h compared to that in the WT hepatocytes (Figure 5C)

Second, the human L02 cell line was treated with ^10^Panx or probenecid, which are two different inhibitors of the PANX1 channel. These two inhibitors suppressed the function of PANX1 but not its expression, as shown in Figure 5D,G. Western blotting analysis showed a substantial increase in cleaved‐IL‐33 production after LPS stimulation, and this expression peaked at 12 h, while the inhibition of PANX1 using ^10^Panx or probenecid substantially impaired cleaved‐IL‐33 synthesis at various time points after 4 h (Figure 5D–F). Collectively, these results suggested that PANX1 was indispensable for IL‐33 synthesis in hepatocytes after LPS administration.

The protein levels of components of the NLRP3‐caspase‐1 pathway were also analyzed. The NLRP3 and caspase‐1 p20 levels were decreased in LPS‐stimulated cells 4 and 8 h, respectively, after treatment with the PANX1 inhibitor (Figure 5D,H,I). P2X7 expression was decreased after the inhibition of PANX1 (Figure 5L), while P2X2 and P2X4 expressions were mostly not significantly altered (Figure 5J,K). The protein levels of cleaved IL‐18 and cleaved IL‐1β were significantly altered beginning at 4 h after treatment with the PANX1 inhibitor (Figure 5D,M,N). These data suggest that the NLRP3‐Caspase‐1 pathway was suppressed by PANX1 inhibition. Furthermore, P2X7, but not P2X2 or P2X4, was involved in PANX1‐dependent IL‐33 expression in hepatocytes after LPS stimulation.

### 3.7 The ATP‐P2X7 pathway regulated hepatic PANX1‐IL‐33 signalling in endotoxemia *in vitro*

**FIGURE 6:**
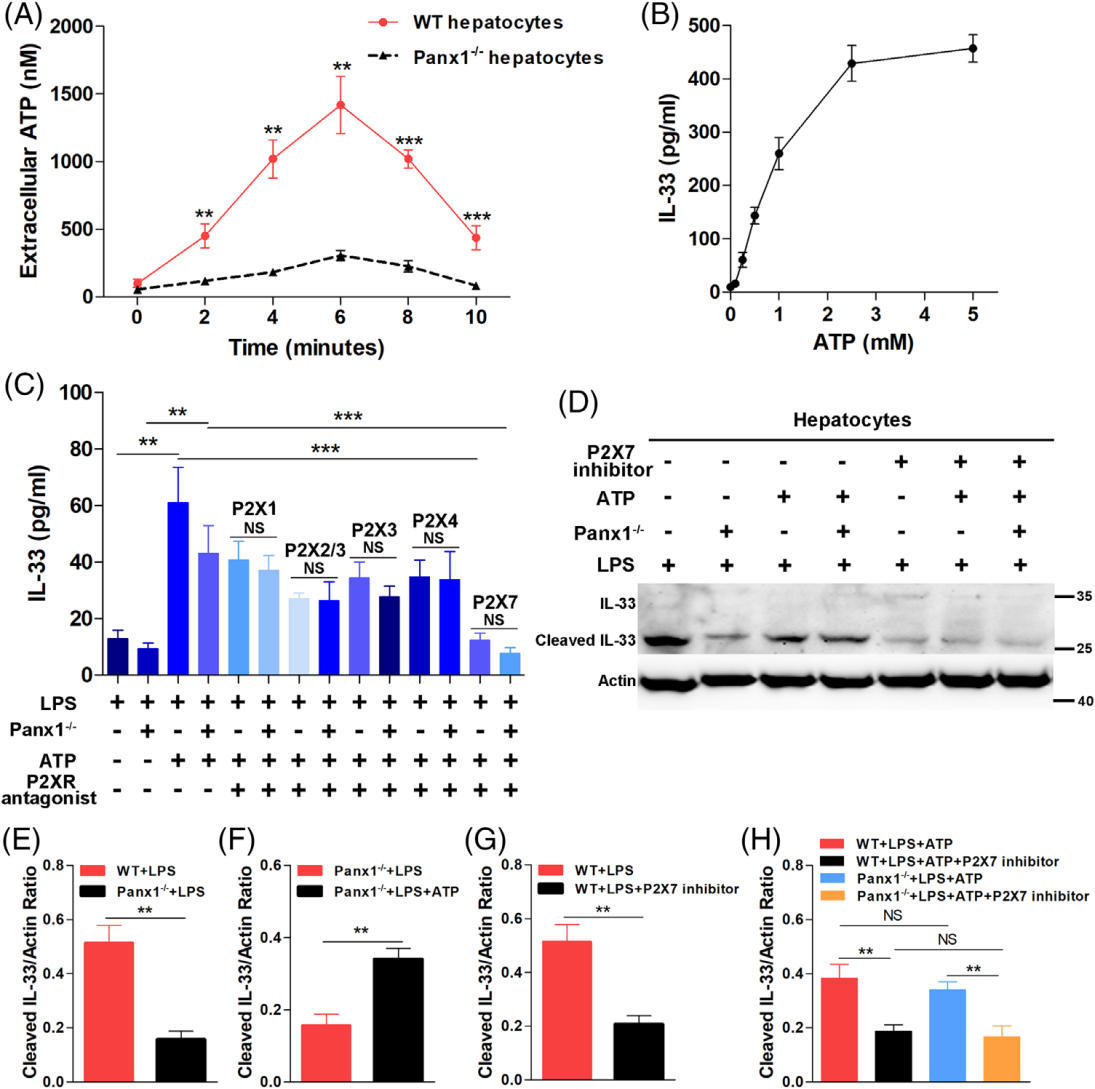
The ATP/P2X7 pathway regulated the hepatic PANX1–IL‐33 axis in endotoxemia *in vitro*. (A) Extracellular adenosine triphosphate (ATP) concentrations in the supernatants of primary WT hepatocytes and Panx1^−/−^ hepatocytes at various time points after lipopolysaccharide (LPS) stimulation (1 μg/ml) (*n *= 5 per group). (B) ATP induced IL‐33 secretion by wild‐type (WT) hepatocytes stimulated with LPS (1 μg/ml) for 12 h (*n* = 4). (C) IL‐33 levels in the supernatants of LPS‐primed (1 μg/ml) primary WT and Panx1^−/−^ hepatocytes treated with ATP (0.25 mM), P2X1 inhibitor (10 μM), P2X2/3 inhibitor (50 μM), P2X3 inhibitor (50 μM), P2X4 inhibitor (10 μM), or P2X7 inhibitor (50 μM) alone or in combination for 12 h (*n* = 4 per group). (D–H) Immunoblots and quantification of IL‐33 expression in LPS‐primed (1 μg/ml) primary WT and Panx1^−/−^ hepatocytes stimulated with ATP (0.25 mM), P2X7 inhibitor (50 μM) or both for 12 h (*n* = 4 per group). **p < 0.01 and ***p < 0.001 by Student's t‐test or ANOVA

Unlike the other two IL‐1 family cytokines, IL‐1β and IL‐18, IL‐33 is inactivated rather than activated by caspases.^41^ The mechanism by which PANX1 controls IL‐33 expression is unknown. PANX1 is a hemichannel that modulates cellular ATP release. Extracellular ATP has been shown to induce IL‐33 secretion by specific cells.^42^, ^43^ We analyzed the local ATP levels in the supernatants of primary hepatocyte cultures after LPS stimulation. As shown in Figure 6A, significantly lower extracellular ATP concentrations were observed in the supernatants of Panx1^−/‐^ hepatocytes than in the supernatants of WT hepatocytes at various time points after LPS administration, and the peak ATP concentration was approximately 1400 nM. Next, we confirmed that IL‐33 release by LPS‐primed hepatocytes could be induced by ATP stimulation (Figure 6B). A positive correlation between the ATP concentration and IL‐33 levels at 12 h was observed (Figure 6B). We chose the relatively low concentration of 0.25 mM ATP to rescue IL‐33 expression in hepatocytes because high levels of ATP substantially increased IL‐33 secretion, and IL‐33 synthesis would therefore be undetectable.^44^

The ELISA results showed that ATP treatment (0.25 mM) significantly increased the IL‐33 levels in the supernatants of both WT and Panx1^−/‐^ LPS‐primed primary hepatocytes at 12 h (Figure 6C). Furthermore, P2X antagonists were utilized to block ATP signal transduction and further study the role of ATP in PANX1‐mediated IL‐33 synthesis and secretion. Among the 5 studied P2XR antagonists (P2X1 inhibitor: NF‐449; P2X2/3 inhibitor: RO‐3; P2X3 inhibitor: NF110; P2X4 inhibitor: 5‐BDBD; and P2X7 inhibitor: KN‐62), the P2X7 inhibitor significantly suppressed IL‐33 secretion by both WT and Panx1^−/−^ LPS‐primed primary hepatocytes in the presence of ATP (Figure 6C). Western blotting showed that ATP rescued cleaved‐IL‐33 synthesis in Panx1^−/−^ primary hepatocytes stimulated with LPS at 12 h (Figure 6D–F). The P2X7 inhibitor also significantly inhibited cleaved‐IL‐33 synthesis in WT hepatocytes (Figure 6D,G). Additionally, both in WT and Panx1^−/−^ hepatocytes, cleaved‐IL‐33 production in the presence of ATP was markedly suppressed by the P2X7 inhibitor (Figure 6D,H). Based on these *in vitro* results, PANX1 regulated IL‐33 synthesis and secretion in LPS‐stimulated hepatocytes via the ATP‐P2X7 pathway.

### 3.8 ATP‐P2X7 pathway regulated the hepatic PANX1‐IL‐33 axis in endotoxemia *in vivo*

**FIGURE 7:**
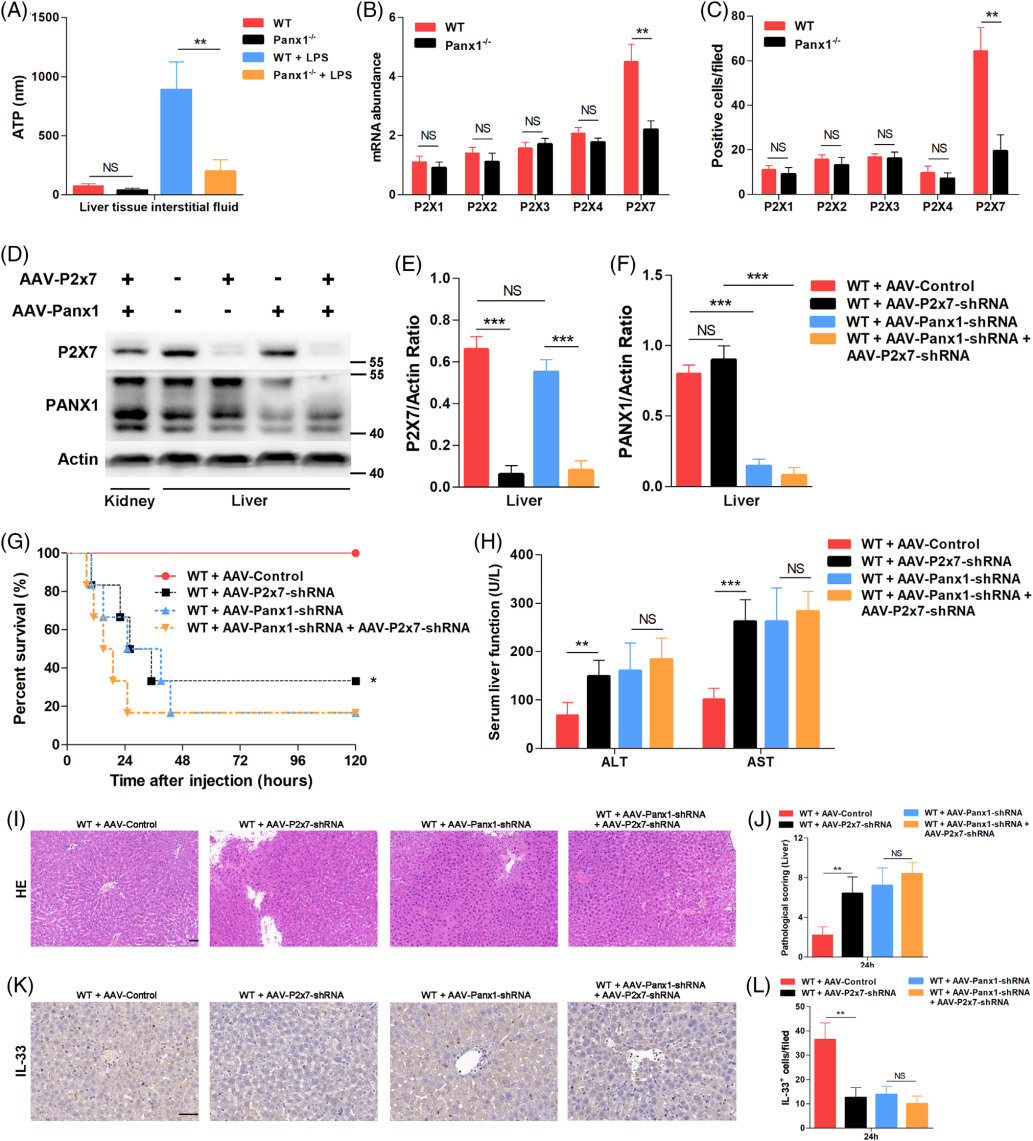
The ATP‐P2X7 pathway regulated the hepatic PANX1–IL‐33 axis in endotoxemia *in vivo*. (A) Adenosine triphosphate (ATP) concentrations in liver tissue interstitial fluid from wild‐type (WT) and Panx1^−/−^ mice at 24 h after injection with 15 mg/kg lipopolysaccharide (LPS) (*n* = 5 mice per group). (B) mRNA levels of P2xs in wild‐type (WT) and Panx1^−/−^ mice at 24 h after injection with 15 mg/kg LPS (*n* = 4 per group). (C) Quantification of IHC staining for P2Xs in liver tissue samples from WT and Panx1^−/−^ mice at 24 h after injection with 15 mg/kg LPS. The quantified results are the average of 10 images per mouse (*n* = 5 mice per group). (D–F) Immunoblots and quantifications of P2X7 and PANX1 expression in the liver and kidney of WT mice treated with adeno‐associated virus (AAV)‐Panx1‐shRNA, AAV‐P2x7‐shRNA or both at 2 weeks. (G) Survival (*n* = 6 mice per group) and (H) serum alanine aminotransferase (ALT) and aspartate aminotransferase (AST) levels (n = 5 mice per group) of control mice and mice treated with AAV‐P2x7‐shRNA, AAV‐Panx1‐shRNA, or both at 24 h after injection with 15 mg/kg LPS. (I and J) Representative images of hematoxylin and eosin staining and pathology scoring (scale bar = 50 μm) (*n* = 5 mice per group). (K and L) Representative images and quantification of IHC staining of IL‐33 in the liver tissue samples of control mice and mice treated with AAV‐P2x7‐shRNA, AAV‐Panx1‐shRNA, or both at 24 h after injection with 15 mg/kg LPS. The quantified results are the average of 10 images per mouse (scale bar = 50 μm) (*n* = 5 mice per group). **p < 0.01 and ***p < 0.001 by Student's t‐test or ANOVA

To determine the mechanism by which PANX1 regulates IL‐33 production *in vivo*, we analyzed the local ATP levels in the liver tissue interstitial fluid of mice injected with LPS. As shown in Figure 7A, the Panx1^−/−^ mice had a significantly lower ATP concentration in their liver tissue interstitial fluid than the WT mice 12 h after LPS administration. Next, we measured the mRNA levels of P2Xs in mouse liver specimens. Consistent with the *in vitro* results, the P2x7 mRNA levels, but not the mRNA levels of other P2Xs, were increased in the WT mice compared with the Panx1^−/−^ mice (Figure 7B). IHC results also showed that there were more P2X7‐positive hepatocytes in the WT mice than in the Panx1^−/−^ mice (Figure 7C and Figure S6C).

To study the crosstalk between P2X7 and PANX1 *in vivo*, AAV carrying P2x7‐specific shRNA and AAV carrying Panx1‐specific shRNA were used to knock down P2x7 expression, Panx1 expression or P2x7 and Panx1 expression in a liver‐specific manner. The hepatic rather than renal expression levels of P2X7 and PANX1 were significantly decreased after knockdown, which was confirmed by Western blotting (Figure 7D–F). As shown in Figure 7G, liver‐specific P2X7 knockdown significantly impaired survival after the LPS challenge. However, the liver‐specific Panx1‐knockdown mice were all susceptible to LPS challenge, regardless of whether P2x7 expression was knocked down. The ALT level, AST levels (Figure 7H), and HE staining results (Figure 7I,J) at 24 h after LPS injection showed that P27‐knockdown mice had more serious liver damage, while there were no significant differences in the Panx1‐knockdown mice. IL‐33 IHC staining showed that P2X7 knockdown significantly suppressed IL‐33 expression in hepatocytes 24 h after LPS stimulation *in vivo* (Figure 7K,L). Again, no difference was observed after P2X7 expression was knocked down in liver‐specific Panx1‐knockdown mice. These results suggested that the ATP‐P2X7 pathway regulated the hepatic PANX1‐IL‐33 axis in endotoxemia *in vivo*.

### 3.9 IL‐33 rescued LPS‐induced endotoxemia and liver injury, which increased liver Treg numbers and attenuated the cytokine storm

**FIGURE 8:**
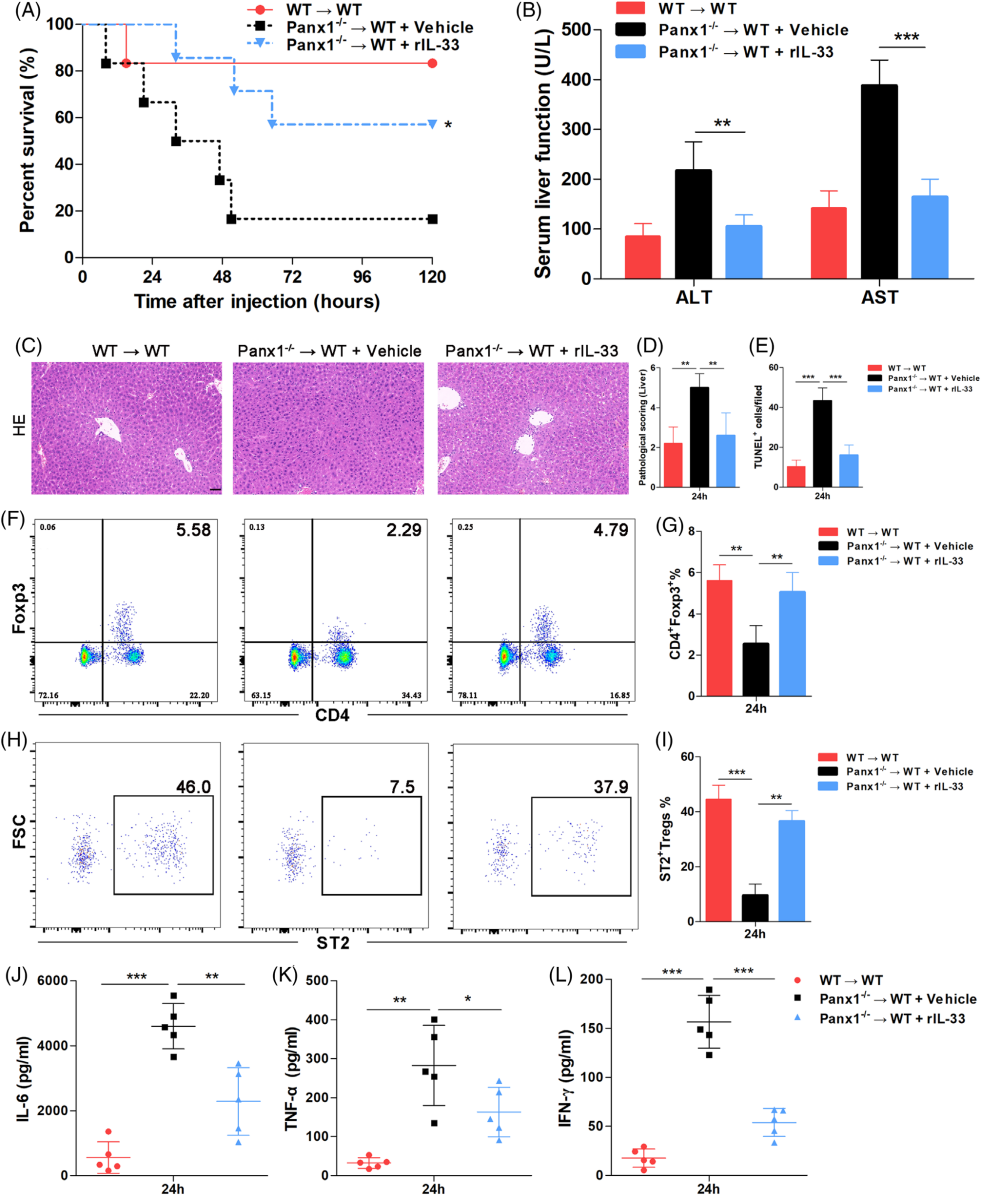
rIL‐33 rescued LPS‐induced endotoxemia by increasing liver‐infiltrating ST2^+^ Treg numbers in hepatic PANX1‐deficient mice. (A) Survival and (B) serum alanine aminotransferase (ALT) and aspartate aminotransferase (AST) levels of wild‐type (WT) → WT mice (*n* = 6) and Panx1^−/−^ → WT mice treated with vehicle (*n* = 6) or rIL‐33 (*n* = 7) at 24 h after injection with 15 mg/kg LPS. (C–E) Representative images and quantification of hematoxylin and eosin staining and TUNEL staining of the liver tissue samples of mice from the three groups at 24 h after LPS injection. The quantified results are the average of 10 images per mouse (scale bar = 50 μm) (*n* = 5 mice per group). Proportions of liver‐infiltrating CD4^+^ Foxp3^+^ Tregs (F and G) and ST2^+^ Tregs (H and I) in mice from the three groups at 24 h after injection with 15 mg/kg LPS (*n* = 5 mice per group). Serum levels of IL‐6 (J), TNF‐α (K), and IFN‐γ (L) in mice from the three groups at 24 h after injection with 15 mg/kg LPS (n = 5 mice per group). *p < 0.05, **p < 0.01, and ***p < 0.001 by Student's t‐test, one‐way ANOVA, or log‐rank test

To study whether IL‐33 could alleviate the hyperinflammation caused by PANX1 deficiency during endotoxemia, we pretreated the Panx1^−/−^ → WT mice with recombinant IL‐33 (rIL‐33) 12 h before LPS injection. As shown in Figure 8A, administration of rIL‐33 (2 μg per mouse) significantly improved the survival of the LPS‐primed Panx1^−/−^ → WT mice. Consistent with these findings, increased serum ALT and AST levels were observed in the Panx1^−/−^ → WT mice compared with the WT → WT mice after the LPS challenge, which could be significantly prevented by rIL‐33 administration (Figure 8B). HE and TUNEL staining showed more serious liver injury in the Panx1^−/−^ → WT mice after LPS administration, and this injury could be improved by rIL‐33 pretreatment (Figure 8C–E and Figure S6D).

Next, we assessed the Treg population in mouse livers. The FACS results showed that there were fewer liver‐infiltrating Tregs in the Panx1^−/−^ → WT mice than in the WT → WT mice; however, these Treg numbers were significantly increased by rIL‐33 treatment (Figure 8F and G). To study the effect of rIL‐33 on Tregs, we measured the expression level of the ST2 receptor in these liver‐infiltrating Tregs. As shown in Figure 8H and I, fewer ST2^+^ Tregs were observed in the Panx1^−/−^ → WT mice, but these numbers could be significantly increased by rIL‐33 injection. Next, we examined circulating cytokine levels to study the immune status of mice. The high levels of IL‐6, TNF‐α, and IFN‐γ caused by hepatic PANX1 deficiency in endotoxemia were significantly decreased by rIL‐33 treatment (Figure 8J‐L). These data suggested that IL‐33 could attenuate the LPS‐induced endotoxemia and liver injury caused by hepatic PANX1 deficiency by increasing the numbers of liver‐infiltrating ST2^+^ Tregs and alleviating the early cytokine storm.

## 4 DISCUSSION

**FIGURE 9:**
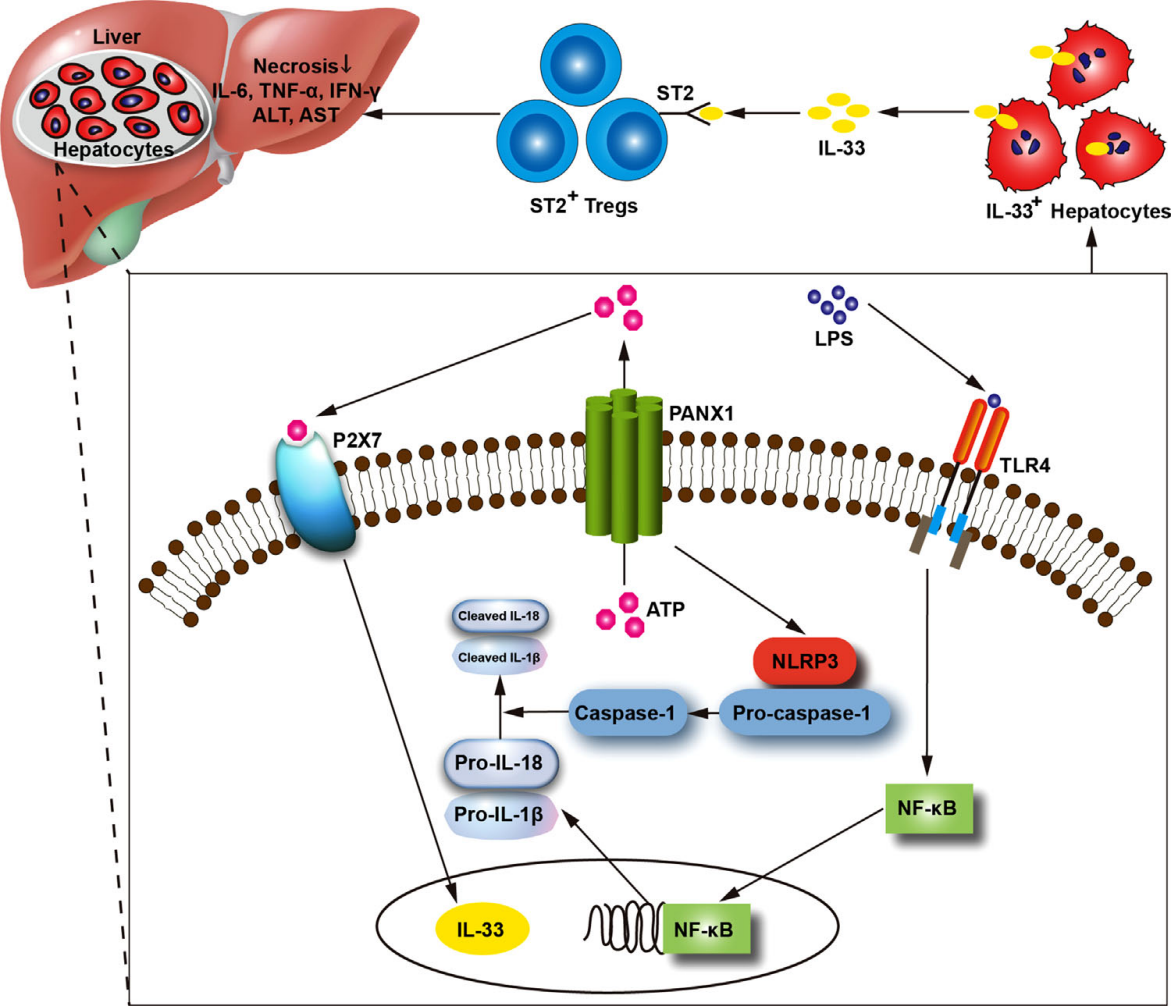
Schematic of the mechanism by which the hepatic PANX1‐IL‐33 axis is regulated by the ATP‐P2X7 pathway and targets ST2^+^ Tregs to attenuate LPS‐induced endotoxemia

In the present study, we showed a protective role of the hepatic PANX1‐IL‐33 axis in LPS‐induced endotoxemia, which supplemented our previous research.^9^ In LPS‐induced endotoxemia, hepatic PANX1 controlled IL‐33 synthesis and secretion via the ATP‐P2X7 pathway, thus targeting ST2^+^ Tregs and promoting the resolution of hyperinflammation (Figure 9).

Bacterial sepsis is a leading cause of death in patients who undergo LT.^5^ Sepsis is characterized by systemic and hyperinflammatory states. Cytokine storm is the main cause of organ injury and death. In contrast, to live bacterial infection, resolution of uncontrolled hyperinflammation rather than pathogen elimination is the key to host recovery from LPS‐induced endotoxemia. This study showed that adaptive immunity, including regulatory cytokines and cells, plays a critical role in LPS‐induced endotoxemia, while innate immunity plays a vital role in MRSA infection in our previous study.^9^ Furthermore, studies have reported a lack of currently available methods for reducing the incidence of sepsis in LT recipients.^4^ Therefore, studying the underlying mechanism and identifying new therapeutic targets in sepsis are of great importance.

In human studies, we investigated the PANX1‐IL‐33 axis in patients with sepsis. To eliminate the potential influence of ischemia‐reperfusion during LT, we enrolled patients who developed sepsis one week to six months after LT in this study, and we obtained serum samples from the control group and the sepsis group at matched time points. Moreover, the control group and sepsis group showed highly similar baseline characteristics, including donor cause of death, cold ischemia time and other donor characteristics. In addition, all the patients with a liver injury who were enrolled in this study underwent biopsy to eliminate the possibility of rejection. Next, based on *in vivo* studies using Panx1^−/−^ mice and *in vitro* studies using two types of hepatocytes, we demonstrated that hepatic PANX1 positively regulated IL‐33 expression. Next, in animal experiments, we determined the protective effect of the hepatic PANX1‐IL‐33 axis on LPS‐induced liver injury using a mouse orthotopic LT model. Consistent with the results of previous studies,^24^, ^25^ PANX1 inhibition or knockout substantially increased the mortality of animals treated with LPS. Although many studies have been performed, controversy persists regarding whether PANX1 exerts a protective or harmful effect on inflammation.^26^, ^45^, ^46^, ^47^, ^48^, ^49^ In some cases of sterile inflammation, PANX1 is required for NLRP3 activation and the subsequent production of proinflammatory cytokines that exacerbate liver damage.^46^, ^47^, ^50^, ^51^, ^52^, ^53^ In contrast to sterile inflammation, low IL‐1β or IL‐18 levels were not detected in Panx1^−/−^ mice with LPS‐induced endotoxemia.

Pyroptosis plays critical roles in LPS‐induced sepsis, including via canonical and noncanonical pathways, toll‐like receptor 4 (TLR4)‐dependent and TLR4‐independent pathways,^54^, ^55^ Gasdermin D (GSDMD) and beyond‐GSDMD‐mediated pathways.^56^ Kang et al. reported that knockout of GSDMD attenuated pyroptosis and protected mice from sepsis.^56^ Hepatocyte pyroptosis was also associated with liver injury and prognosis in sepsis.^57^ In contrast to the *in vivo* results, Caspase‐1, IL‐1β and IL‐18 were activated in hepatocytes after LPS administration *in vitro*, while PANX1 inhibition significantly reduced pyroptosis. Moreover, IL‐33 levels were positively associated with pyroptosis *in vitro*. However, complete disruption of PANX1 function *in vivo* impaired host immune defences against virulence factors, which led to decreased numbers of activated immune cells and clinical sepsis.^23^, ^25^ Therefore, LPS induced the production of higher levels of these proinflammatory cytokines and more severe liver injury in Panx1^−/−^ mice than in WT mice. In LPS‐induced endotoxemia, since there is no bacterial proliferation, resolution rather than persistent induction of hyperinflammation via IL‐33‐Tregs is vital to protect organs. In a mouse orthotopic LT model, we found that the hepatic PANX1‐IL‐33 axis increased circulating and liver‐infiltrating Treg numbers and attenuated excessive inflammation, which protected the liver from LPS‐induced injury. However, pyroptosis *in vivo* was not studied directly with or without rIL‐33 treatment. Through HE staining and TUNEL staining, we demonstrated that IL‐33‐Tregs attenuated liver injury, which suggested that IL‐33 targeting Tregs might reduce pyroptosis.

IL‐33 normally functions as an alarmin that is released by necrotic cells. In our study, this process was dependent on PANX1. Our results showed two forms of IL‐33, full‐length IL‐33 (IL‐33_fl_) and cleaved IL‐33 (Figure 5D). Cayrol et al. designed an experiment and demonstrated that the IL‐33_fl_ precursor had biological activity and, importantly, that it was still in a full‐length form at the end of the bioassays.^31^ Unlike IL‐1β and IL‐18 but similar to IL‐1α, IL‐33 has biological activity as a full‐length molecule.^31^ Moreover, Lefrançai et al. showed 25/26 kDa cleavage products of IL‐33 using translated full‐length human IL‐33_1–270_, and their mutagenesis studies indicated that the 25/26 kDa cleavage product corresponds to two distinct mature forms of IL‐33, namely, IL‐33_79–270_ and IL‐33_72–270_.^58^ These two cleavage products had intact IL‐1‐like cytokine domain and consequently had markedly increased biological activity compared to IL‐33_fl_.^31^, ^58^ According to these studies, we considered that the main band observed at approximately 25 kDa in our study was cleaved IL‐33_72–270_ and bioactive. Among Panx1^−/−^ hepatocytes, greater numbers of apoptotic cells were accompanied by decreased IL‐33 expression compared to WT hepatocytes. Various studies have reported protective roles for IL‐33 in liver injury induced by concanavalin A,^45^ ischemia‐reperfusion,^59^ virus infection,^60^ and nonalcoholic steatohepatitis.^61^ The mechanisms underlying the protective effects of IL‐33 identified in these studies were partially elucidated by evidence showing that the Th2 response is promoted, Treg numbers are increased, and caspase‐3 activation is suppressed by the IL‐33‐ST2 axis. As shown in the study by Nascimento et al., IL‐33 increases the expansion of Tregs and induces immunosuppression following sepsis.^39^ Consistent with these results, we observed uncontrolled hyperinflammation, more substantial thymic involution, decreased Treg numbers, increased CD19^+^ B cell numbers and increased circulating and local hepatic IL‐33 levels in Panx1^−/−^ mice compared with WT mice. Furthermore, our findings indicated that IL‐33 played a protective role against LPS‐induced endotoxemia through adaptive immunity in addition to innate immunity, which supplemented previous studies.^9^, ^34^

Next, we observed significantly reduced ATP concentrations in the supernatants of Panx1^−/−^ hepatocytes. ATP can induce IL‐33 mRNA and protein expression in airway epithelial cells,^62^ human corneal epithelial cells,^44^ mast cells,^63^ and bronchial smooth muscle cells.^64^ Therefore, we examined whether IL‐33 synthesis and secretion by hepatocytes could be induced by ATP. Indeed, ATP induced IL‐33 secretion by LPS‐stimulated hepatocytes in a concentration‐dependent manner. LPS administration also increased IL‐33 synthesis in hepatocytes, a process that could be largely impaired by PANX1 inhibition or knockout. However, without ATP, LPS alone did not induce IL‐33 secretion by WT hepatocytes *in vitro*. This result occurred due to the *in vitro* experimental conditions, under which the ATP concentration was too low to induce IL‐33 release. After treatment with high levels of exogenous ATP, both IL‐33 synthesis and secretion were restored. We also investigated whether its receptors, P2Xs, altered IL‐33 expression to confirm the role of ATP. P2Xs are widely expressed by various cell types and are involved in many signal transduction pathways. In terms of inflammation, Kim et al.^65^ reported that the Panx1‐P2X4 pathway played a critical role in HCV‐infected hepatocytes. Some other studies reported that P2X7 was critical in LPS‐induced inflammation,^63^, ^66^, ^67^ which was consistent with this study. In our previous study,^9^ we screened different P2X subtypes and demonstrated that P2X2 was involved in MRSA infection. In this study, we also screened the P2X family; however, we found that classical P2X7 rather than other P2Xs regulated the PANX1‐IL‐33 axis. Specifically, P2X1, P2X2/3, P2X3, P2X4, and P2X7 antagonists were used in experiments that assessed IL‐33 synthesis and secretion because of the lack of other selective P2X antagonists. We showed that the P2X7 antagonist almost completely blocked IL‐33 synthesis and secretion. Of note, P2X7 expression was mildly decreased at some time points after ^10^Panx treatment *in vitro*. However, in both Panx1‐knockout hepatocytes and WT hepatocytes treated with the P2X7 antagonist, IL‐33 synthesis was markedly suppressed, and there was no statistical significance in IL‐33 production. Consequently, we cannot determine whether the P2X7 inhibitor had less of an effect in the presence or absence of PANX1.

In brief, we demonstrated that PANX1 regulates IL‐33 production via the ATP‐P2X7 pathway. First, we showed that IL‐33 production was dependent on PANX1 in two kinds of hepatocytes by treating the cells with two PANX1 inhibitors or genetically depleting PANX1. PANX1 is an important ATP channel, so we showed that PANX1 deficiency resulted in lower ATP levels. To analyze the role of ATP in PANX1‐dependent IL‐33 production, we applied ATP stimulation and ATP‐P2X pathway inhibitors. ATP stimulation significantly increased IL‐33 release by both WT control cells and PANX1‐deficient cells and rescued IL‐33 synthesis in PANX1‐deficient cells. Next, among the five ATP‐P2X pathway inhibitors we screened, we found that the P2X7 inhibitor was the most effective. P2X7 inhibitor, which blocks ATP signal transduction, markedly blocked IL‐33 synthesis and release in the presence or absence of ATP or PANX1, which meant that P2X7 regulated IL‐33 induction after PANX1‐ATP in the whole pathway.

After we confirmed that the ATP‐P2X7 pathway regulates the PANX1‐IL‐33 axis *in vitro*,*in vivo* studies were performed. We observed decreased ATP concentrations in the liver tissue interstitial fluid of Panx1^−/‐^ mice and decreased P2X7 expression levels compared with the levels of other P2Xs in the liver tissues of Panx1^−/‐^ mice. Next, by injecting AAV‐P2x7‐shRNA and AAV‐Panx1‐shRNA to knockdown hepatic expression of P2X7, PANX1, or both in mice, we demonstrated that P2x7 knockdown had no effect on Panx1‐knockdown mice. These results suggested that P2X7 mediates PANX1‐IL‐33 deficiency‐related liver injury and death in LPS‐induced endotoxemia. Consistently, Kataoka et al. reported that ATP induced IL‐33 expression by stimulating the P2X7 receptor under conditions of GNB infection.^68^ Another study also reported that the P2X7 receptor was involved in IL‐33 production by increasing the concentration of Ca^2+^.^62^ Whether P2X7 can regulate any transcription factor that induces IL‐33 expression, such as CDX2, remains unclear.^69^ The exact mechanism by which P2X7 regulates IL‐33 production remains unclear and represents a topic for future studies. In addition, rIL‐33 pretreatment significantly protected Panx1^−/‐^ → WT mice against LPS‐induced endotoxemia. Liang et al.^60^ and Volarevic et al.^45^ also reported that the injection of rIL‐33 protects against liver injury. Furthermore, we showed that hepatic IL‐33 protects against LPS‐induced endotoxemia by increasing liver‐infiltrating ST2^+^ Treg numbers and promoting the early resolution of excessive inflammation.

Collectively, we reported low PANX1 expression in donor grafts prior to LT and decreased IL‐33 levels after LT in clinical patients with sepsis. Using mouse orthotropic LT and mouse primary hepatocyte models, we proposed that the main mechanism by which PANX1 attenuates LPS‐induced liver injury is by promoting ATP‐P2X7 pathway‐dependent IL‐33 synthesis and secretion, which increases liver‐infiltrating ST2^+^ Treg numbers and attenuates excessive inflammation. Based on our findings, the PANX1‐IL‐33 axis may represent a new target for treating sepsis after LT.

## CONFLICT OF INTEREST

The authors declare no conflict of interest.

## Supporting information



Supporting Information

Supporting Information

Supporting Information

Supporting Information

Supporting Information

Supporting Information
