# Supplementary figures and images for: Hepatic pannexin‐1 mediates ST2+ regulatory T cells promoting resolution of inflammation in lipopolysaccharide‐induced endotoxemia
Source: Clin Transl Med. 2022 May 20;12(5):e849. doi: 10.1002/ctm2.849 (PMC9121315; doi:10.1002/ctm2.849)

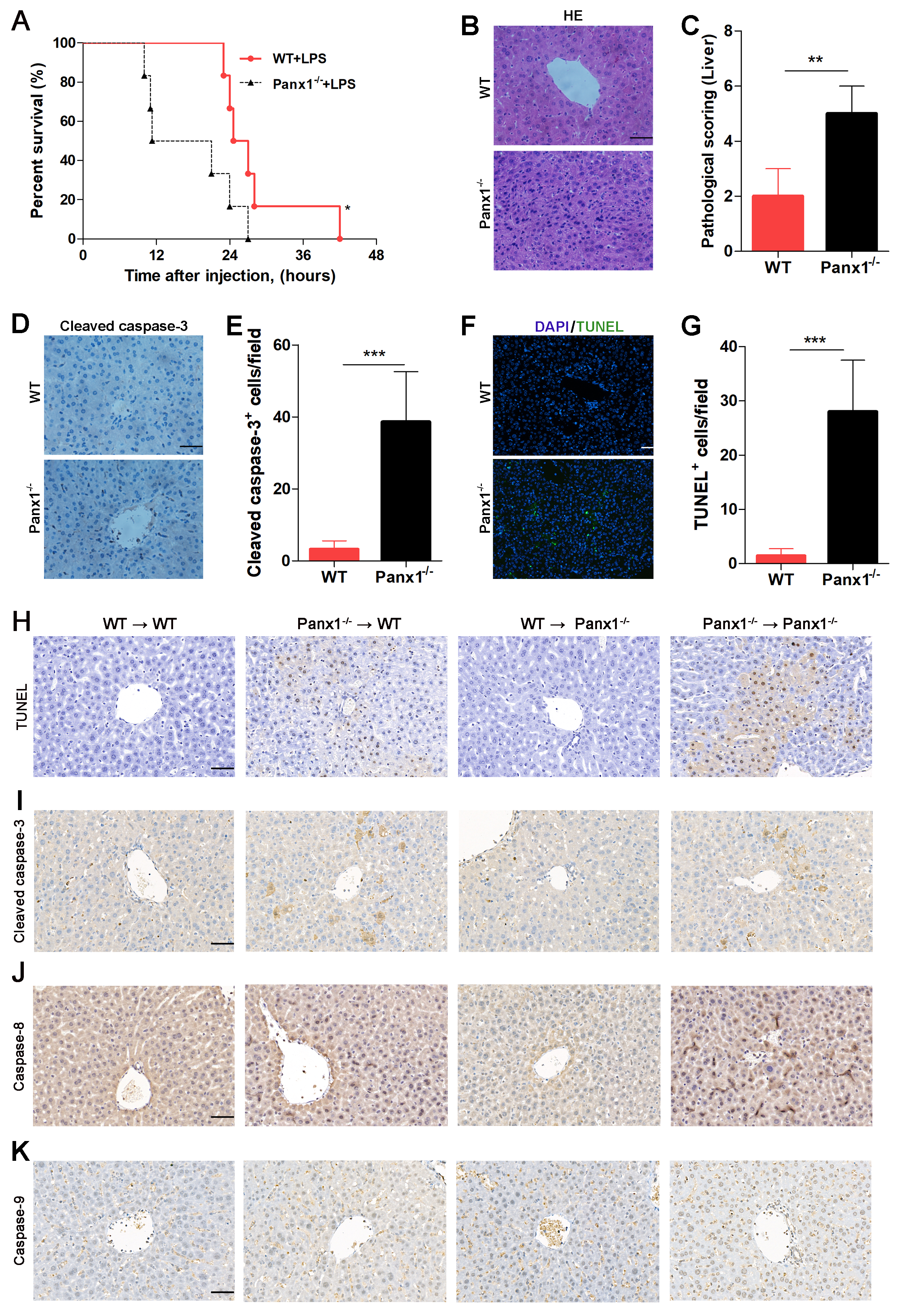

Supplement: Supplementary file 1 — Supporting Information [file CTM2-12-e849-s003.tif]

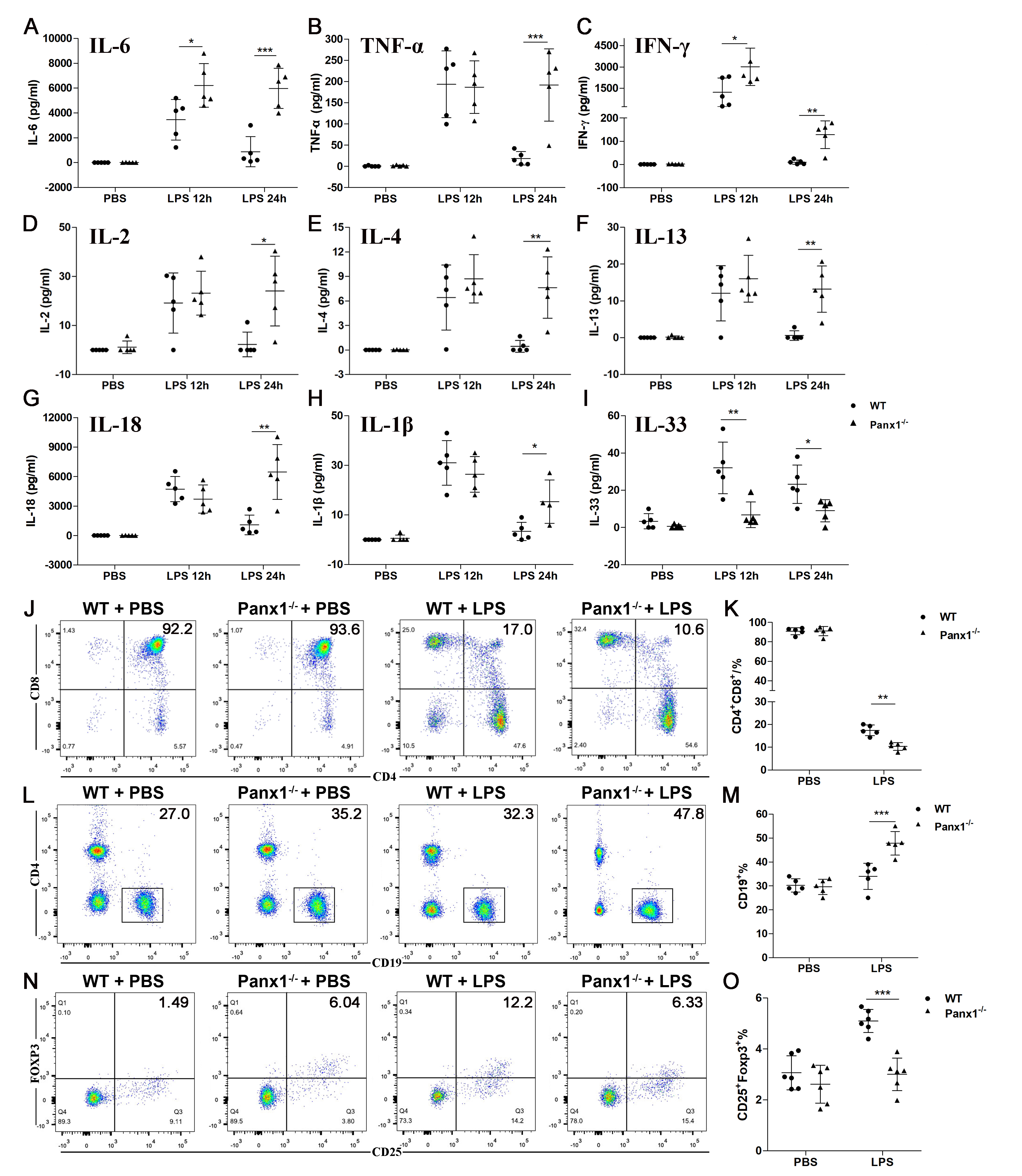

Supplement: Supplementary file 2 — Supporting Information [file CTM2-12-e849-s004.tif]

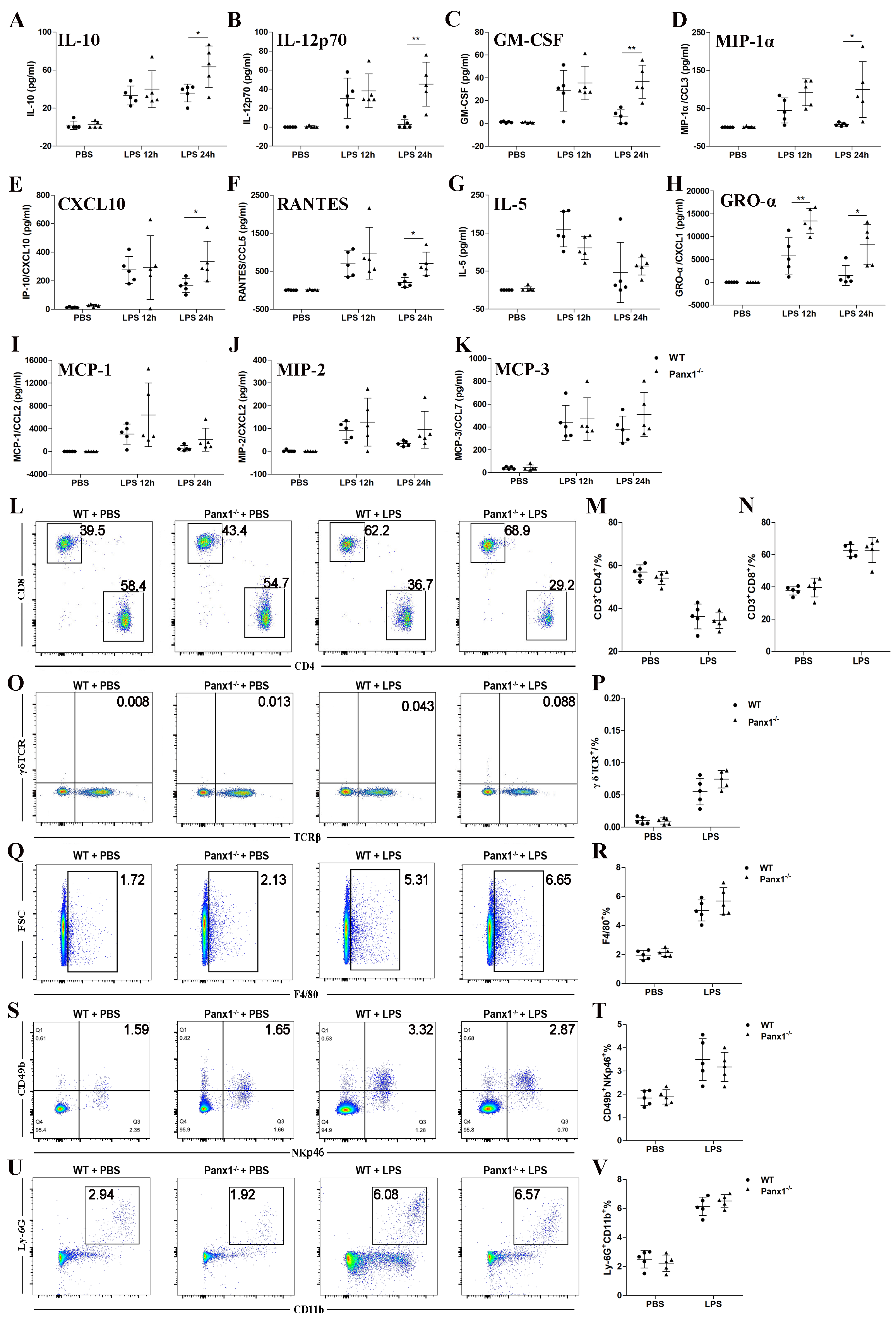

Supplement: Supplementary file 3 — Supporting Information [file CTM2-12-e849-s006.tif]

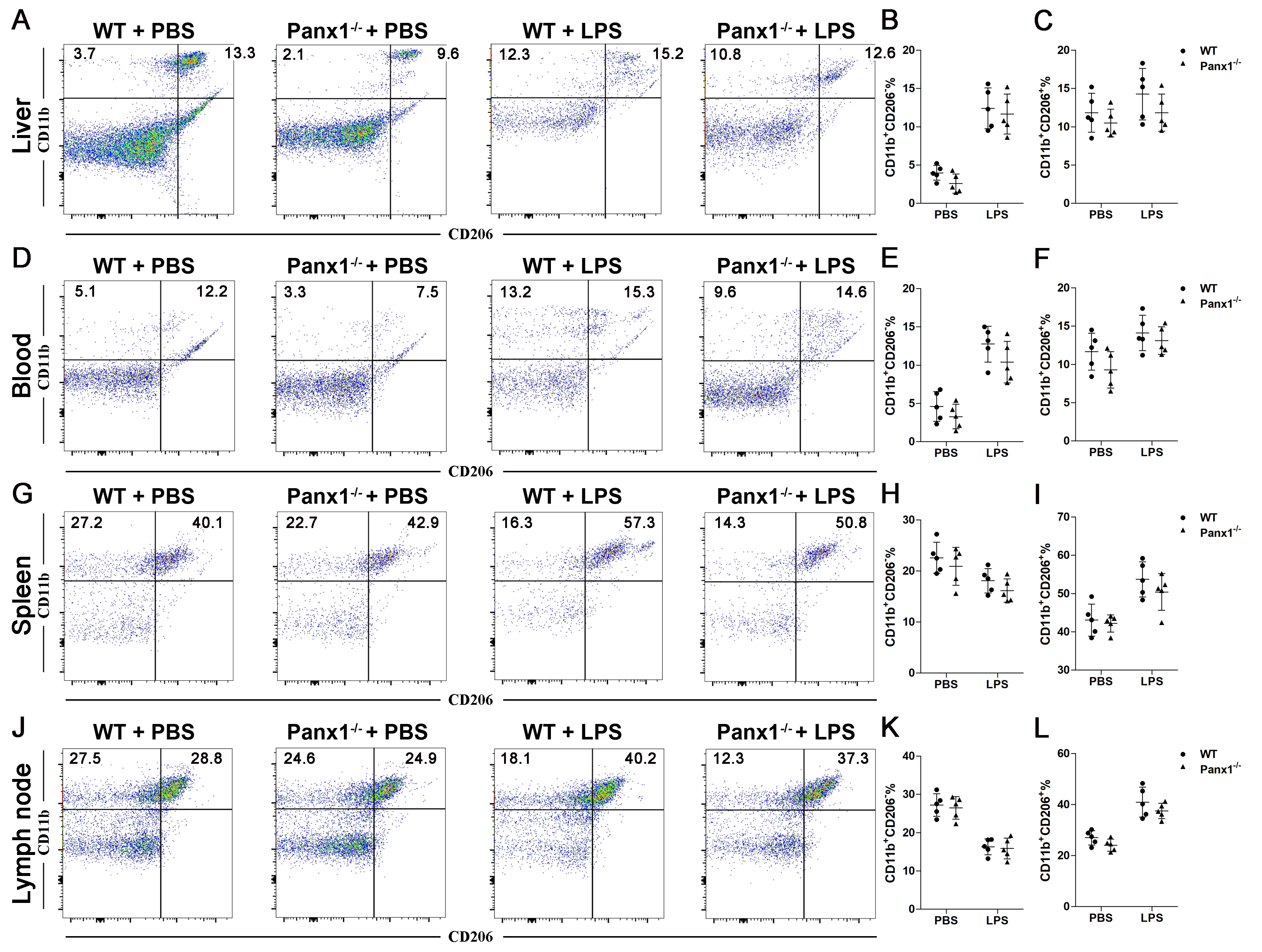

Supplement: Supplementary file 4 — Supporting Information [file CTM2-12-e849-s005.tif]

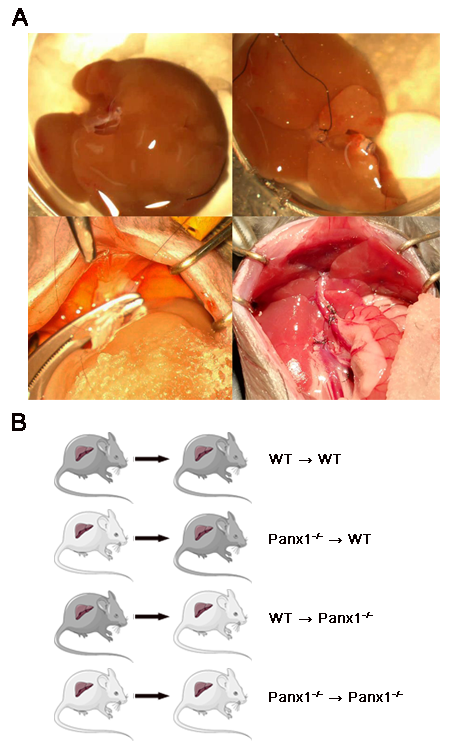

Supplement: Supplementary file 5 — Supporting Information [file CTM2-12-e849-s002.tif]

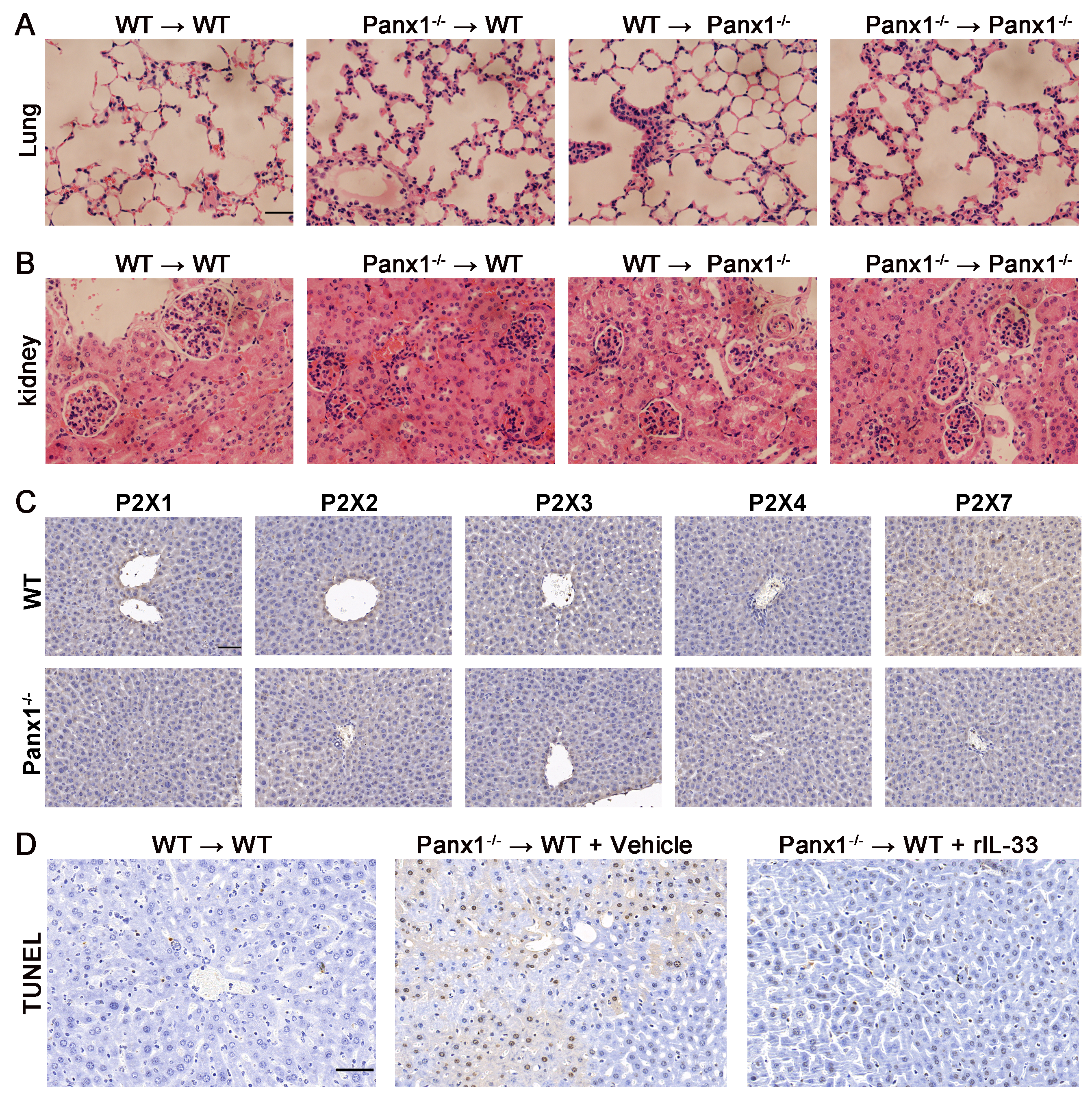

Supplement: Supplementary file 6 — Supporting Information [file CTM2-12-e849-s001.tif]
